# Supplementary material for: “I Go up to the Edge of the Valley, and I Talk to God”: Using Mixed Methods to Understand the Relationship between Gender-Based Violence and Mental Health among Lebanese and Syrian Refugee Women Engaged in Psychosocial Programming
Source: Int J Environ Res Public Health. 2021 Apr 23;18(9):4500. doi: 10.3390/ijerph18094500 (PMC8123009; doi:10.3390/ijerph18094500)
Supplement: Supplementary file 1 [file ijerph-18-04500-s001.zip › Qualitative Tools/FGD Guide Community members GWI Abaad_ARA.docx.pdf]

العلاقة بين العنف القائم على نوع الجنس والصحة النفسية بين الناجيات في لبنان

دليل مناقشة مجموعة التركيز: أفراد المجتمع

المقدمة

ملاحظة مهمة بخصوص نوع الجنس:

نظراً إلى موضوع مناقشات مجموعات التركيز هذه، يوصى بفصل النساء والرجال خلالها. قبل البدء بمناقشة مجموعة التركيز، يتعين ضمان الحصول الموافقة المسبقة.

معلومات هامة ومقدمة[1]

مرحباً، اسمي ____ وأنا أعمل في ______. كنا نعمل في ____ (المنطقة) من أجل ____ (نوع العمل) طوال ____ (الفترة الزمنية). حالياً، نحن نتحدث مع أشخاص نعتقد أنهم يفهمون ديناميات واحتياجات المرأة في هذا المجتمع. في هذه المناقشة، أود أن أسألك عن المشاكل المختلفة التي يواجهها الناس في المجتمع، وعن وصولهم إلى الخدمات وعن إمكانية الوصول إلى تلك الخدمات. أود أيضاً أن أسأل كيف يتعامل الناس مع هذه المشاكل. ستبدأ المناقشة بأسئلة عامة حول الخدمات وإمكانية الوصول إليها، وتنتقل إلى أسئلة أكثر تحديداً حول العنف داخل مجتمعك وحول تأثيره عليك شخصياً وعلى رفاهك النفسي مع الاحتياجات في مجال الصحة النفسية في الوصول إلى الخدمات. هدفنا هو التعلم من معلوماتك وخبرتك، كي نكون أكثر قدرة على تقديم الدعم.

1. هدفنا هو التعلم من معرفتك وخبرتك، كي نكون أكثر قدرة على تقديم الدعم.
2. لا يمكننا أن نعد بتقديم الدعم لك في مقابل هذه المقابلة. نحن هنا فقط لطرح الأسئلة والتعلم من تجاربك.
3. يمكنك الاختيار بين المشاركة أو عدم المشاركة.
4. إذا اخترت المشاركة في هذه المناقشة، يمكنني أن أؤكد لك أنه لن يتم مشاركة معلوماتك إلا مع الأشخاص الموجودين في هذه الغرفة.
5. على الرغم من أننا نشجع مشاركتك، إلا أنه يمكنك الخروج من المناقشة في أي وقت.
6. هل لديك أي أسئلة؟ هل ترغب في المشاركة في المناقشة؟

1. نعم
2. لا

معلومات حول مناقشة مجموعة التركيز

التاريخ: __/___/______ **اسم الميسر:**

1 الفريق المرجعي التابع للجنة الدائمة المشتركة بين الوكالات المعني بالصحة النفسية والدعم النفسي الاجتماعي في حالات الطوارئ. التقييم التشاركي الثاني: تصورات أفراد المجتمع مع معرفة عميقة بالمجتمع. في: دليل تقييم الصحة النفسية والدعم النفسي والاجتماعي للفريق المرجعي التابع للجنة الدائمة المشتركة بين الوكالات، الذي سيصدر قريباً. وقد تم استخدام هذه الأداة هنا بإذن من الفريق المرجعي التابع للجنة الدائمة المشتركة بين الوكالات.

مدوّن الملاحظات (إن وجد):	موقع مناقشة مجموعة التركيز:
عدد المشاركين في هذه المجموعة (المجموع):	المنطقة (المناطق) التي يعيش فيها المشاركون:

مدة مناقشة مجموع التركيز (بالدقائق):

القسم أ. أسئلة عامة حول وضع المرأة في [الموقع]

أ. 1 ما المشاكل التي تشكل أكبر مصدر للقلق بالنسبة للنساء في [الموقع]؟ يرجى إدراج أكبر عدد من المشاكل التي يمكنك التفكير فيها.

ملاحظة: عندما لا يكون عدد الإجابات الطلوبة محدداً، استمر في تشجيع المستجيب على تقديم المزيد من الإجابات. على سبيل المثال بعد أن يعدد المستجيب بعض المشاكل ويتوقف عن الكلام، يمكنك أن تسأل:

"ما نوع المشاكل الأخرى التي تواجهها النساء في [الموقع]؟ يرجى ذكر أكبر عدد من المشاكل التي يمكنك التفكير بها". عندها، قد يذكر المستجيب بعض المشاكل الأخرى. وتستمر بعد ذلك بطرح السؤال إلى أن يتوقف المستجيب عن إعطاء أي إجابات أخرى.

بعد اكتمال القائمة، يجب أن تطلب وصفاً موجزاً لكل مشكلة مدرجة بحيث يمكن ملء الجدول التالي (الجدول 1).

الجدول 1. قائمة المشاكل (من أي نوع)[2]

المشكلة	الوصف
**1.1.1**	
**1.1.2**	
**1.1.3**	
**1.1.4**	
**1.1.5**	

[2] اللجنة الدائمة المشتركة بين الوكالات.

	1.1.6
	1.1.7
	1.1.8
	1.1.9
	1.1.10
	1.1.11
	1.1.12

**1.1.13**	
**1.1.14**	
**1.1.15**	
**1.1.16**	
**1.1.17**	
**1.1.18**	
**1.1.19**	
**1.1.20**	

أ. 2 **الميسِّر:** أنظر إلى الإجابات على السؤال أ.1 واتبع التعليمات الواردة أدناه لاختيار مشاكل الصحة النفسية والمشاكل النفسية الاجتماعية تحديداً. اشرح للمشاركين بأنك تقوم بذلك.

حدد المشاكل ذات الصلة بشكل خاص من منظور الصحة النفسية/الوضع النفسي والاجتماعي، مثل: المشاكل المتعلقة بالعلاقات الاجتماعية (العنف المنزلي والمجتمعي، إساءة معاملة الأطفال، انفصال الأسرة) ؛ (ب) المشاكل المتعلقة بما يلي:

- المشاعر (على سبيل المثال الشعور بالحزن أو الخوف)؛
- التفكير (على سبيل المثال القلق)؛ أو
- السلوك (على سبيل المثال شرب الكحول).

انسخ هذه المشاكل في الجدول 1.2 أدناه[3].

الجدول 1.2 قائمة بمشاكل الصحة النفسية/المشاكل النفسية الاجتماعية الأسباب المحتملة

[3] اللجنة الدائمة المشتركة بين الوكالات

The Global Women's Institute
THE GEORGE WASHINGTON UNIVERSITY

1.2.1

1.2.2

1.2.3

1.2.4

1.2.5

1.2.6

1.2.7

1.2.8

1.2.9

متى ملأت الجدول (كميسر)، اسأل عن المشاكل الثلاث ذات الأولوية، وميِّزها أو ضع خطاً تحتها بلون ماركر مختلف.

أ.3 ما هي الأنواع المختلفة من العنف التي تواجهها المرأة في مجتمعك؟

أ.4 ما هي برأيك العلاقة بين العنف القائم على نوع الجنس والصحة النفسية بالنسبة للنساء في مجتمعك؟

أ.4 أ إذا وجد المشاركون صعوبة في الإجابة عن هذا السؤال، فقم برسم مخطط انسيابي يساعدهم على تحديد أنواع مختلفة من العنف القائم على نوع الجنس وعلاقتها بالصحة النفسية. ***(استخدم ذلك فقط إذا لم يستطع المشاركون الإجابة على السؤال أ.4 بسهولة، لأن هذا يستغرق وقتاً)***

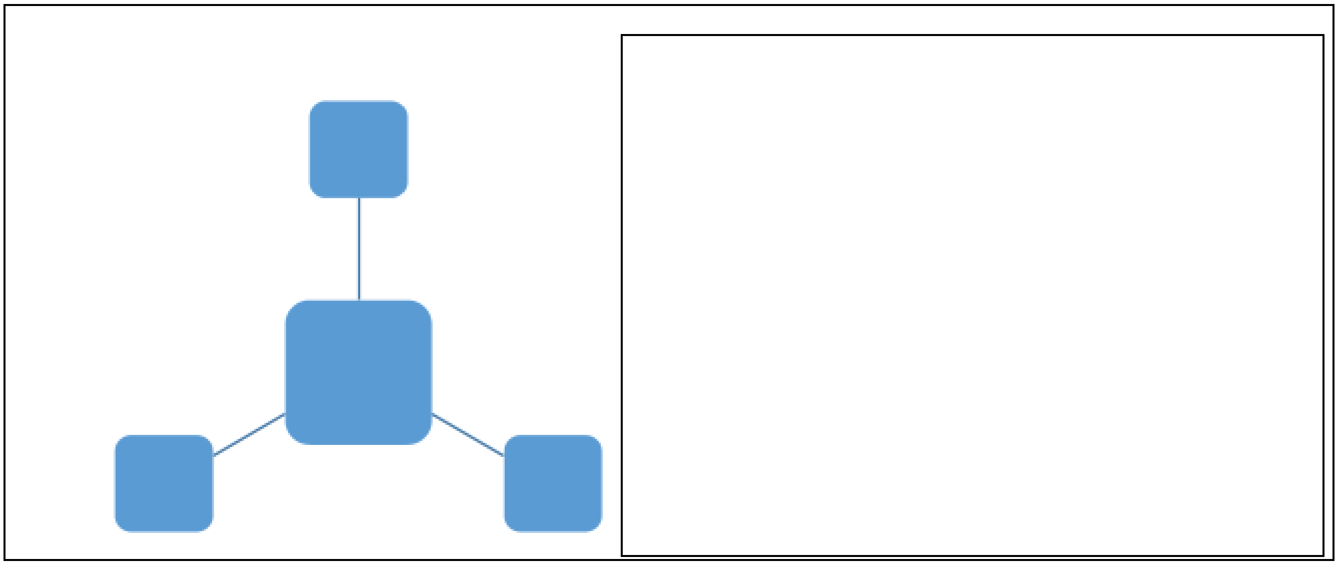

مثال:

يمكن استخدام هذا الرسم البياني لإظهار الأنواع المختلفة من العنف القائم على نوع الجنس وتأثيرها على الجوانب المختلفة من الصحة النفسية. ومن أجل رسمه، اسأل أولاً ما هي أنواع العنف القائم على نوع الجنس الموجودة في المجتمع، وقم بتصنيفها (على سبيل المثال، الضرب والخنق هما نوعان من عنف جسدي). ثم اسألهم عن الجوانب المختلفة للصحة النفسية (مثل

النوع من العنف القائم على نوع الجنس أن يؤثر على [جانب من جوانب الصحة النفسية]؟

أحد أنواع العنف القائم على نوع الجنس وارسم جوانب الصحة النفسية كأقسام متفرعة منه واسأل: كيف يمكن لهذا

الصحية، والرضا العام عن الحياة، والتعامل مع الشدائد، وما إلى ذلك). ارسم العديد من الرسوم البيانية وضع في وسطها

السلوك، والمشاعر، والثقة بالنفس، والقدرة على القيام بالمهام اليومية، والقدرة على الحفاظ على العلاقات

القسم ب. الخدمات وإمكانية الوصول إلى الخدمات

ب.1 الميسِّر: بناءً على ما سبق، أعطِ مثالاً عن امرأة قد تكون تعاني من القضايا المذكورة أعلاه. أعطِ هذه المرأة اسماً، وعمراً، وحدد نوع المنطقة التي تقيم فيها منزلها، ثم أدرج المشاكل التي قد تكون تواجهها (استناداً إلى الأمثلة المذكورة أعلاه). ثم اسأل المشاركين: ما هي الخدمات المتوفرة في منطقتك والتي ستقوم بإحالة هذه المرأة إليها من أجل معالجة هذه القضايا؟ يمكن أن تكون هذه الخدمات غير رسمية (الأسرة، الأصدقاء، المنشآت التعليمية غير الرسمية، المراكز المجتمعية)، أو رسمية (المستشفيات، مراكز تقديم الخدمات، العيادات).

مثال: فرح *(24* عاماً، من سوريا، تعيش في مخيم غير رسمي*)*، أخبرتك سراً بأنها حزينة لأن شقيقها عاطل عن العمل ولا يستطيع إعالة أسرته. كما أخبرتك أنها لم تتمكن من الوصول إلى الخدمات الطبية لمعالجة مرضها المزمن، ولم تستطع هي وأسرتها تحمل تكاليف الرعاية الطبية. ما هي الخدمات التي توصي بها لها في المنطقة؟ أمثلة أخرى عن المشاكل التي قد تطرأ: عدم الحصول على ما يكفي من الغذاء/ الماء /الملابس الدافئة، والمشاكل الزوجية التي تنتهي بالعنف، وعدم الوصول إلى التعليم، وترك الأطفال في المنزل، وعدم القدرة على الذهاب لرؤية الأصدقاء/السعي للحصول على الدعم النفسي والاجتماعي بسبب العنف الذي يمارسه الشريك، إلخ

ب.1 أ إلى من تقدَّم هذه الخدمات ، وبأي طريقة؟ (احتمالات: العيادات، المراكز المجتمعية، الزيارات المنزلية، صراحةً). قد تكون أنواع الخدمات كما يلي: الخدمات الصحية، والدعم النفسي والاجتماعي، وإدارة الحالة، والخدمات القانونية، والحماية/الأمن، والتوعية/الوقاية، والدعم العاطفي الأساسي، والأنشطة الجماعية، والمعونة الغذائية، والمأوى، والتعليم، والنظافة، إلخ.

كيف: أطلب من المشاركين رسم خريطة أساسية للمنطقة (المناطق) التي يعملون فيها، وإدراج الخدمات المختلفة المقدمة في تلك المنطقة على الخريطة. يجب أن تُظهر الملصقات الملونة نوع الخدمة ومكانها. ويجب أن تشير ملصقات تدوين الملاحظات تحتها إلى الأشخاص الذين تستهدفهم هذه الخدمات والأشخاص الذين يتمتعون بفرصة أكبر بالوصول إليها.

ب.2 ما هي أفضل طريقة لتقديم/إيصال المعلومات إلى [المرأة في القصة أعلاه] وغيرها من النساء، حول الخدمات في مجتمعك؟

ب.2 أ هل كان مقدمو الخدمات فعالين في تقديم هذه المعلومات للنساء؟ والناجيات من العنف القائم على نوع الجنس؟

ب.2 أ 1 كيف يجب برأيك نشر هذه المعلومات بين مجموعات مستهدفة مختلفة (رجال، نساء، مراهقون، ذوو إعاقات، أشخاص لديهم احتياجات محددة في مجال الصحة النفسية)؟

ب.3 هل تشعر بالارتياح عند السعي للحصول على هذه الخدمات؟ هل تشعر أن الناجيات من العنف القائم على نوع الجنس يشعرن بالارتياح عند السعي للحصول عليها؟

ما نوع الحواجز التي تمنع النساء بشكل عام من الوصول إلى هذه الخدمات؟ (احتمالات: الحواجز الجغرافية والأمنية، الحواجز العائلية/الاجتماعية، والخدمات غير الكافية، إلخ.). أثناء إجابة المشاركين على السؤالين ب.4 وب.4 أ، املأ الجدول أدناه، ثم اسأل عن أهم 3 حواجز، مع تمييزها/تسطيرها بلون مختلف.

ب.4 أ ما الذي يمكن القيام به لتحسين ذلك؟

الجدول 1.4: لائحة الحواجز التي تمنع الناجيات من العنف القائم على نوع الجنس من الوصول إلى الخدمات مع اعتبارات في مجال الصحة النفسية	الحلول المحتملة
1.2.1	
1.2.2	
1.2.3	
1.2.4	
1.2.5	
1.2.6	
1.2.7	
1.2.8	
1.2.9	
1.2.10	

القسم ج. أسئلة محددة حول الناجيات من العنف القائم على نوع الجنس واللواتي لديهن احتياجات محددة في مجال الصحة النفسية

ج.1 ما هي المشاكل التي تشكل أكبر مصدر للقلق بالنسبة للناجيات من العنف القائم على نوع الجنس واللواتي لديهن احتياجات محددة في مجال الصحة النفسية في [الموقع]؟ يرجى إدرج أكبر عدد من المشاكل التي يمكنك التفكير فيها. الميسر: اكتب هذه المشاكل في هذا الجدول. قد يشمل العنف القائم على نوع الجنس العنف الجسدي (مثل الضرب والدفع والطعن وغيرها من الأعمال الضارة التي تؤذي المرأة جسدياً) والعنف العاطفي/النفسي (مثل الإذلال وحرمان المرأة من رؤية عائلتها/أصدقائها وتخويفها وعزلها) والعنف الجنسي (أداء أفعال جنسية من أي نوع ضد إرادة الشخص أو دون موافقته)، و/أو العنف الاقتصادي (على سبيل المثال منع المرأة من الوصول إلى الموارد النقدية أو اتخاذ أي قرارات تتعلق بالمال، وعدم السماح للنساء بالحصول على المال لتلبية الاحتياجات الأساسية مثل الماء أو الطعام، أو الاحتياجات الشخصية). قد تشمل الاحتياجات في مجال الصحة النفسية أعراض الاكتئاب والقلق والاضطرابات الذهانية/العصبية، والإجهاد، والإجهاد الضار، وغيرها من الأعراض التي قد تحتاج المرأة التي تعاني منها إلى دعم أو تدخل.

المشكلة	الوصف
1.1.1	
1.1.2	
1.1.3	
1.1.4	
1.1.5	
1.1.6	
1.1.7	
1.1.8	
1.1.9	
1.1.10	
1.1.11	
1.1.12	
1.1.13	
1.1.14	
1.1.15	
1.1.16	
1.1.17	
1.1.18	
1.1.19	
1.1.20	

ج.1 أ هل تختلف هذه المشاكل عن المشاكل التي تواجهها نساء أُخريات؟

ج.1 ب إذا كانت تختلف، فكيف؟ وهل هناك حاجة إلى تقديم خدمات محددة لهذه المجموعة المستهدفة؟ (إذا كان الأمر كذلك، فكر في نوع الخدمات، وتوافرها).

ج.1 ب 1 إذا كانت هذه الخدمات متوفرة، فهل النساء الناجيات من العنف القائم على نوع الجنس اللواتي لديهن احتياجات محددة في مجال الصحة النفسية يشعرن بالراحة عند السعي للحصول على هذه الخدمات؟

ج.2 الميسِّر: بناءً على ما سبق، أعطِ مثالاً عن امرأة قد تكون تعاني من القضايا المذكورة أعلاه. أعطِ هذه المرأة اسماً، وعمراً، وحدد نوع المنطقة التي تقيم فيها منزلها، ثم أدرج المشاكل التي قد تكون تواجهها (استناداً إلى الأمثلة المذكورة أعلاه). ثم اسأل المشاركين: ما هي الحواجز التي قد تمنع هذه المرأة بشكل خاص، وغيرها من النساء الناجيات من العنف القائم على نوع الجنس واللواتي لديهن احتياجات في مجال الصحة النفسية، من الوصول إلى الخدمات التي تحدثنا عنها؟

مثال: لمى (37 عاماً، من لبنان، تعيش في منطقة ريفية)، أخبرتك سراً بأنها تعاني من نوبات هلع كل أسبوع ولا تستطيع مغادرة السرير معظم الأيام. أخبرتك أن والدها أصبح رجلاً غاضباً، وأنه يدفعها ويهينها أمام عائلتها لأنها غير متزوجة. تشعر لمى بعدم الأمان عند السير في الشوارع وحدها لأنها تعرضت لاعتداء جنسي ومضايقة من جيرانها، ولأنها تتعرض للسخرية بسبب إصابتها بمتلازمة داون. ما هي الخدمات التي توصي بها لها في المنطقة؟ أمثلة أخرى عن المشاكل التي قد تطرأ على بعض النساء: عدم الوصول إلى الدعم النفسي والاجتماعي لمعالجة حالات الضيق، والمشاكل الزوجية التي تنتهي بالعنف، وعدم الوصول إلى المال لتلبية الاحتياجات الأساسية أو الشخصية، وعدم القدرة على الذهاب لرؤية الأصدقاء/السعي للحصول على الدعم النفسي والاجتماعي بسبب العنف الذي يمارسه الشريك، إلخ.

ج.1 أ ما هي بعض طرق العمل للتغلب على هذه العقبات؟

ج.1 ب ما الذي يمكن أن يفعله على وجه التحديد قادة المجتمع/مقدمو الخدمات لتحقيق ذلك؟

القسم د: آليات المواجهة في المجتمع

د.1 أخيراً، سنتحدث عن بعض آليات التكيف المستخدمة في مجتمعك. اذكر أدناه مشاكل الصحة النفسية/المشاكل النفسية والاجتماعية المذكورة في الجدول 1.2 (الميسر: ابقِ هذا الجدول مرئياً، واقرأ الإجابات ذات الأولوية إذا لزم الأمر)، بالإضافة إلى آلية التكيف الأكثر شيوعاً بالنسبة للنساء

الناجيات من العنف القائم على نوع الجنس واللواتي لديهن احتياجات في مجال الصحة النفسية.

الجدول 1.4 آليات التكيف[4]

مشاكل الصحة النفسية/المشاكل النفسية والاجتماعية (كما هي مذكورة في 1.2)	آلية التكيف	هل طريقة التكيف هذه مفيدة؟
1.2.1	3.2.1	نعم/لا
1.2.2	3.2.2	نعم/لا
1.2.3	3.2.3	نعم/لا
1.2.4	3.2.4	نعم/لا

4 اللجنة الدائمة المشتركة بين الوكالات.

1.2.5		3.2.5	نعم/لا
1.2.6		3.2.6	نعم/لا

اختتم المناقشة[5]

- أشكر المشاركين على وقتهم ومساهماتهم.
- ذكِّر المشاركين بأن الغرض من هذه المناقشة هو فهم احتياجات وشواغل النساء والفتيات بشكل أفضل منذ الأزمة.
- ذكِّر المشاركين بموافقتهم على السرية.
- ذكِّر المشاركين بعدم مشاركة المعلومات أو أسماء المشاركين الآخرين مع أفراد آخرين في المجتمع.
- اسأل المشاركين إذا كان لديهم أسئلة.
- إذا رغب أحد في التحدث على انفراد، فأجب بأن الميسر وأمين السر سيكونان متاحين بعد الاجتماع.

[5] لجنة الإنقاذ الدولية (2011). مجموعة أدوات التقييم الخاصة بلجنة الإنقاذ الدولية.
