# Supplementary material for: “I Go up to the Edge of the Valley, and I Talk to God”: Using Mixed Methods to Understand the Relationship between Gender-Based Violence and Mental Health among Lebanese and Syrian Refugee Women Engaged in Psychosocial Programming
Source: Int J Environ Res Public Health. 2021 Apr 23;18(9):4500. doi: 10.3390/ijerph18094500 (PMC8123009; doi:10.3390/ijerph18094500)
Supplement: Supplementary file 1 [file ijerph-18-04500-s001.zip › Qualitative Tools/FGD Guide Service Providers Community Leaders GWI Abaad.pdf]

Relationship between GBV and Mental Health among Female Survivors in Lebanon

FGD Guide: Service providers/community leaders

Introduction

Important note:

Before commencing the focus group discussion, ensure informed consent is received.

Important information and introduction[1]

Hello, my name is _____ and I work for _____. We have been working in ___ (area) to _____ (type of work) for ___ (period). Currently, we are talking to people who we believe know a lot about services available in this region. In this interview I would like to ask you about various problems people in the community have, their access to service and the accessibility of those services. I would also like to ask how people deal with these problems. The discussion will start with general questions about services and accessibility, and move into more specific questions about violence within your community and how it affects you personally and your mental wellbeing with MH needs accessing services. Our aim is to learn from your knowledge and experience, so that we will be better able to provide support.

1. Whatever you choose to answer will not affect your access to services now or in the future, by ABAAD or any other service provision agency
2. Your participation is voluntary—you are free to take part or not
3. If you choose to partake in this discussion, I can assure you that your information will not leave this room, and will only be shared with the people in this room.
4. Your participation should take between 45-60 minutes
5. Though your participation is encouraged, you can step out of the discussion at any point
6. Do you have any questions? Would you like to partake in the discussion?

1. Yes

2. No

FGD Information

**Date:** __ / __ / _____	**Facilitator name:**
**Note-taker (if available):**	**Location of FGD:**
**Number of participants in this group (total):**	**Area(s) where participants live:**
**Length of FGD (in minutes):**	

[1] IASC Reference Group on Mental Health and Psychosocial Support in Emergency Settings. Participatory Assessment II: Perceptions by community members with in-depth knowledge of the community. In: IASC Reference Group Mental Health and Psychosocial Support Assessment Guide, forthcoming. This tool has been reproduced here with permission from the IASC Reference Group.

A.1 What issues are of greatest concern for women in [location]? List as many problems as you can think of

Note: When using free listing, you keep on encouraging the respondent to give more answers. For example after the respondent has listed a few problems and remains silent, you could ask:

"What other kind of problems do women have in [location]? Please list as many problems that you can think of." The respondent may now list a few more problems. You would then continue with the question until the respondent gives no more answers.

After the list is completed, you should ask for a short description of each problem listed so that the following table (Table 1) can be completed.

TABLE 1. List of problems (of any kind)[2]

Problem	Description
1.1.1	
1.1.2	
1.1.3	
1.1.4	
1.1.5	
1.1.6	
1.1.7	
1.1.8	
1.1.9	
1.1.10	
1.1.11	
1.1.12	
1.1.13	
1.1.14	
1.1.15	
1.1.16	
1.1.17	
1.1.18	

[2] IASC.

1.1.19	
1.1.20	

A.2 **Facilitator**: look at the responses to question A.1 and follow the instructions below to select mental health and psychosocial problems specifically. Explain to the participants that you are doing this.

Select those problems which are especially relevant from a mental health / psychosocial perspective, such as:

Problems related to social relationships (domestic and community violence, child abuse, family separation); and

(b) Problems related to:

- Feelings (for example feeling sad or fearful);
- Thinking (for example worrying); or
- Behavior (for example drinking).

Copy these into Table 1.2 below. [3]

TABLE 1.2 LIST OF MENTAL HEALTH/PSYCHOSOCIAL PROBLEMS	POSSIBLE CAUSES
1.2.1	
1.2.2	
1.2.3	
1.2.4	
1.2.5	
1.2.6	
1.2.7	
1.2.8	
1.2.9	
1.2.10	

Once you (as a facilitator) have filled these out, ask about the top three priority problems, and highlight or underline them in a different color marker.

[3] IASC.

Part B Services and service accessibility

B.1 Facilitator: based on the above, give an example of a woman who might be experiencing the above issues. Give this woman a name, an age, and geographical residence/house type, then list the issues that she might have (based on examples given above). Then, ask the participants: what services are available in your area would you refer this woman to in order to address these issues?

> *Example: Farah (24, from Syria, living in informal tent settlement), confided in you that she was distressed because her brother didn't have a job and couldn't provide for the family. She also told you that she has not been able to access medical services for her chronic illness, and she and her family couldn't afford medical care. What services would you recommend to her in the area? Other examples of issues that might arise: lack of access to enough food/water/warm clothes, inter-marital issues resulting in violence, lack of access to education, leaving children at home, inability to go see friends/seek psychosocial support because of abusive partner, etc.*

B.1a To whom are these services being provided, and in what way? (Probe: clinics, community centers, home visits, openly). Types of services might be health, PSS, case management, legal, protection/security, sensitization/prevention, basic emotional support, group activities, food aid, shelter, education, hygiene, etc. Have participants draw do a social mapping activity.

> *How to: ask participants to draw basic map of the area(s) they work in, placing the different services offered in that area on the map. Colored stickers should show the type of service, and where it's located. Sticky notes under them should note who those services are targeted toward, and who has most access to them.*

B.2 How can [woman from story above] get information about available services? Is it easily available?

B.2a Have you found your methods of distributing information about services to be effective in reaching women? GBV survivors?

B.1a1 If so, how do you distribute this information to different target groups (men, women, adolescents, people with disabilities, people with specific MH needs)?

B.2a2 If not, what can be done to improve this?

B.3 Do you feel that women are comfortable seeking these services? GBV survivors? Why or why not?

B.4 What kind of barriers exist for women in general to access these services? (Probe: geographical, security barriers, familial/societal barriers, etc.). As participants give answers to B.4 and B.4a, fill out the table below, then ask for the top 3 barriers, highlighting/underlining them in a different color.

B.4a What can be done to improve this?

TABLE 1.4 LIST OF BARRIERS TO SERVICES FOR GBV SURVIVORS WITH MH CONSIDERATIONS	POSSIBLE SOLUTIONS
1.2.1	
1.2.2	
1.2.3	
1.2.4	
1.2.5	
1.2.6	
1.2.7	
1.2.8	
1.2.9	
1.2.10	

Part C Specific questions about GBV survivors with specific mental health needs

C.1 What issues are of greatest concern for women GBV survivors with specific MH needs in [location]? List as many problems as you can think of. Facilitator: write these problems in this table. GBV can include physical violence (e.g. hitting, pushing, stabbing, other harmful acts that physical harm a woman), emotional/psychological violence (e.g. humiliating, banning women from seeing their family/friends, intimidation, and isolation), sexual violence (performing sexual acts *of any kind* against a person's will or without their consent), and/or economic violence (e.g. preventing women from accessing monetary resources or making any decisions related to money, not allowing women money for basic needs such as water or food, or personal needs). Mental health needs may include symptoms of depression, anxiety, psychotic/neurotic disorders, distress, toxic stress, and other symptoms for which women may need support or intervention.

**Problem**	**Description**
1.1.1	
1.1.2	
1.1.3	
1.1.4	
1.1.5	
1.1.6	
1.1.7	
1.1.8	

1.1.9	
1.1.10	
1.1.11	
1.1.12	
1.1.13	
1.1.14	
1.1.15	
1.1.16	
1.1.17	
1.1.18	
1.1.19	
1.1.20	

C.1a Do these differ from the issues that other women face?

C.1b If so, how? Is there a need for specific services for this target group? (If so, probe on what kind of services, and their availability).

C.2 Facilitator: based on the above, give an example of a woman who might be experiencing the above issues. Give this woman a name, an age, and geographical residence/house type, then list the issues that she might have (based on examples given above). Then, ask the participants: what barriers might exist specifically for this woman, and other women GBV survivors with MH needs to access the services we spoke about?

Example: Lama (37, from Lebanon, living in rural area), confided in you that she was experiencing panic attacks every week and can't get out of bed most days. She told you that her father has become an angry man, and that he pushes her and humiliates her in front of her family because she is unwed. Lama feels unsafe walking in the streets alone because she has experienced sexual assault and harassment from her neighbors, and that her down syndrome is a source of ridicule. What services would you recommend to her in the area? Other examples of issues that might arise for some women: lack of access to psychosocial support for distress, inter-marital issues resulting in violence, lack of access to money for basic or personal needs, inability to go see friends/seek psychosocial support because of abusive partner, etc.

C.2a What are some ways to work on overcoming these barriers?

C.2b Do you know of any mechanism already being employed in your region to do so?

C.2c What can be done specifically by community leaders/service providers to do so?

CONCLUDE THE DISCUSSION[4]

- Thank participants for their time and their contributions.
- Remind participants that the purpose of this discussion was to better understand the needs and concerns of women and girls since the crisis.
- Remind participants of their agreement to confidentiality.
- Remind participants not to share information or the names of other participants with others in the community.
- Ask participants if they have questions.
- If anyone wishes to speak in private, respond that the facilitator and secretary will be available after the meeting.

[4] IRC (2011). IRC Assessment ToolKit.
