# Supplementary material for: “I Go up to the Edge of the Valley, and I Talk to God”: Using Mixed Methods to Understand the Relationship between Gender-Based Violence and Mental Health among Lebanese and Syrian Refugee Women Engaged in Psychosocial Programming
Source: Int J Environ Res Public Health. 2021 Apr 23;18(9):4500. doi: 10.3390/ijerph18094500 (PMC8123009; doi:10.3390/ijerph18094500)
Supplement: Supplementary file 1 [file ijerph-18-04500-s001.zip › Qualitative Tools/FGD Guide Service Providers Community Leaders GWI Abaad_ARA.docx.pdf]

أبعاد abaad

The Global Women's Institute
THE GEORGE WASHINGTON UNIVERSITY

العلاقة بين العنف القائم على نوع الجنس والصحة النفسية بين الناجيات في لبنان

دليل مناقشة مجموعة التركيز: مقدمو الخدمات/قادة المجتمع

المقدمة

ملاحظة مهمة:

قبل البدء بمناقشة مجموعة التركيز، يتعين ضمان الحصول الموافقة المسبقة.

معلومات هامة ومقدمة[1]

مرحباً، اسمي ____ وأنا أعمل في ______. كنا نعمل في ____ (المنطقة) من أجل ____ (نوع العمل) طوال ____ (الفترة الزمنية). حالياً، نحن نتحدث مع أشخاص نعتقد أنهم يملكون معلومات كثيرة حول الخدمات المتوفرة في المنطقة. في هذه المقابلة، أود أن أسألك عن المشاكل المختلفة التي يواجهها الناس في المجتمع، وعن وصولهم إلى الخدمات وعن إمكانية الوصول إلى تلك الخدمات. أود أيضاً أن أسأل كيف يتعامل الناس مع هذه المشاكل. ستبدأ المناقشة بأسئلة عامة حول الخدمات وإمكانية الوصول إليها، وتنتقل إلى أسئلة أكثر تحديداً حول العنف داخل مجتمعك وحول تأثيره عليك شخصياً وعلى رفاهك النفسي مع الاحتياجات في مجال الصحة النفسية في الوصول إلى الخدمات. هدفنا هو التعلم من معلوماتك وخبرتك، كي نكون أكثر قدرة على تقديم الدعم.

1. مهما كانت إجابتك فلن تؤثر على وصولك إلى الخدمات التي توفرها أبعاد أو أي وكالة أخرى لتقديم الخدمات، في الوقت الراهن أو في المستقبل.
2. مشاركتك طوعية- يمكنك الاختيار بين المشاركة أو عدم المشاركة.
3. إذا اخترت المشاركة في هذه المناقشة، يمكنني أن أؤكد لك أنه لن يتم مشاركة معلوماتك إلا مع الأشخاص الموجودين في هذه الغرفة.
4. تتراوح مدة المناقشة بين 45 و60 دقيقة.
5. على الرغم من أننا نشجع مشاركتك، إلا أنه يمكنك الخروج من المناقشة في أي وقت.
6. هل لديك أي أسئلة؟ هل ترغب في المشاركة في المناقشة؟

1. نعم
2. لا

أبعاد
abaad

The Global
Women's Institute
THE GEORGE WASHINGTON UNIVERSITY

معلومات حول مناقشة مجموعة التركيز

التاريخ: __/___/______

اسم الميسر:

1 الفريق المرجعي التابع للجنة الدائمة المشتركة بين الوكالات المعني بالصحة النفسية والدعم النفسي الاجتماعي في حالات الطوارئ. التقييم التشاركي الثاني: تصورات أفراد المجتمع مع معرفة عميقة بالمجتمع. في: دليل تقييم الصحة النفسية والدعم النفسي والاجتماعي للفريق المرجعي التابع للجنة الدائمة المشتركة بين الوكالات، الذي سيصدر قريباً. وقد تم استخدام هذه الأداة هنا بإذن من الفريق المرجعي التابع للجنة الدائمة المشتركة بين الوكالات.

أبعاد
abaad

The Global Women's Institute
THE GEORGE WASHINGTON UNIVERSITY

**مدوّن الملاحظات (إن وجد):**	**موقع مناقشة مجموعة التركيز:**
**عدد المشاركين في هذه المجموعة (المجموع):**	**المنطقة (المناطق) التي يعيش فيها المشاركون:**

مدة مناقشة مجموع التركيز (بالدقائق):

القسم أ. أسئلة عامة حول وضع المرأة في [الموقع]

أ. 1 ما هي المشاكل التي تشكل أكبر مصدر للقلق بالنسبة للنساء في [الموقع]؟ يرجى إدرج أكبر عدد من المشاكل التي يمكنك التفكير فيها.

ملاحظة: عندما لا يكون عدد الإجابات الطلوبة محدداً، استمر في تشجيع المستجيب على تقديم المزيد من الإجابات. على سبيل المثال بعد أن يعدد المستجيب بعض المشاكل ويتوقف عن الكلام، يمكنك أن تسأل:

"ما نوع المشاكل الأخرى التي تواجهها النساء في [الموقع]؟ يرجى ذكر أكبر عدد من المشاكل التي يمكنك التفكير بها". عندها، قد يذكر المستجيب بعض المشاكل الأخرى. وتستمر بعد ذلك بطرح السؤال إلى أن يتوقف المستجيب عن إعطاء أي إجابات أخرى.

بعد اكتمال القائمة، يجب أن تطلب وصفاً موجزاً لكل مشكلة مدرجة بحيث يمكن ملء الجدول التالي (الجدول 1).

الجدول 1. قائمة المشاكل (من أي نوع)[2]

المشكلة	الوصف
**1.1.1**	
**1.1.2**	
**1.1.3**	
**1.1.4**	
**1.1.5**	
**1.1.6**	

2 اللجنة الدائمة المشتركة بين الوكالات.

	1.1.7
	1.1.8
	1.1.9
	1.1.10
	1.1.11
	1.1.12

**1.1.13**	
**1.1.14**	
**1.1.15**	
**1.1.16**	
**1.1.17**	
**1.1.18**	
**1.1.19**	
**1.1.20**	

أ. 2 **الميسِّر:** أنظر إلى الإجابات على السؤال أ.1 واتبع التعليمات الواردة أدناه لاختيار مشاكل الصحة النفسية والمشاكل النفسية الاجتماعية تحديداً. اشرح للمشاركين بأنك تقوم بذلك.

حدد المشاكل ذات الصلة بشكل خاص من منظور الصحة النفسية/الوضع النفسي والاجتماعي، مثل: المشاكل المتعلقة بالعلاقات الاجتماعية (العنف المنزلي والمجتمعي، إساءة معاملة الأطفال، انفصال الأسرة) ؛ (ب) المشاكل المتعلقة بما يلي:

- المشاعر (على سبيل المثال الشعور بالحزن أو الخوف)؛
- التفكير (على سبيل المثال القلق)؛ أو
- السلوك (على سبيل المثال شرب الكحول).

انسخ هذه المشاكل في الجدول 1.2 أدناه[3].

[3] اللجنة الدائمة المشتركة بين الوكالات

الجدول 1.2 قائمة الأسباب المحتملة بمشاكل الصحة النفسية/المشاكل النفسية الاجتماعية

1.2.1

1.2.2

1.2.3

1.2.4

1.2.5

1.2.6

1.2.7

1.2.8

1.2.9

1.2.10

متى ملأت الجدول (كميسر)، اسأل عن المشاكل الثلاث ذات الأولوية، وميِّزها أو ضع خطاً تحتها بلون ماركر مختلف.

القسم ب. الخدمات وإمكانية الوصول إلى الخدمات

ب.1 الميسِّر: بناءً على ما سبق، أعطِ مثالاً عن امرأة قد تكون تعاني من المشاكل المذكورة أعلاه. أعطِ هذه المرأة اسماً، وعمراً، وحدد نوع المنطقة التي تقيم فيها منزلها، ثم أدرج المشاكل التي قد تكون تواجهها (استناداً إلى الأمثلة المذكورة أعلاه). ثم اسأل المشاركين: ما هي الخدمات المتوفرة في منطقتك والتي ستقوم بإحالة هذه المرأة إليها من أجل معالجة هذه المشاكل؟

مثال: فرح *(24* عاماً، من سوريا، تعيش في مخيم غير رسمي*)*، أخبرتك سراً بأنها حزينة لأن شقيقها عاطل عن العمل ولا يستطيع إعالة أسرته. كما أخبرتك أنها لم تتمكن من الوصول إلى الخدمات الطبية لمعالجة مرضها المزمن، ولم تستطع هي وأسرتها تحمل تكاليف الرعاية الطبية. ما هي الخدمات التي توصي بها لها في المنطقة؟ أمثلة أخرى عن المشاكل التي قد تطرأ*:* عدم الحصول على ما يكفي من الغذاء*/* الماء */*الملابس الدافئة، والمشاكل الزوجية التي تنتهي بالعنف، وعدم الوصول إلى التعليم، وترك الأطفال في المنزل، وعدم القدرة على الذهاب لرؤية الأصدقاء*/*السعي للحصول على الدعم النفسي والاجتماعي بسبب العنف الذي يمارسه الشريك، إلخ

ب.1 أ إلى من تقدَّم هذه الخدمات ، وبأي طريقة؟ (احتمالات: العيادات، المراكز المجتمعية، الزيارات المنزلية، صراحةً). قد تكون أنواع الخدمات كما يلي: الخدمات الصحية، والدعم النفسي والاجتماعي، وإدارة الحالة، والخدمات القانونية، والحماية/الأمن، والتوعية/الوقاية، والدعم العاطفي الأساسي، والأنشطة الجماعية، والمعونة الغذائية، والمأوى، والتعليم، والنظافة، إلخ.

كيف: أطلب من المشاركين رسم خريطة أساسية للمنطقة (المناطق) التي يعملون فيها، وإدراج الخدمات المختلفة المقدمة في تلك المنطقة على الخريطة. يجب أن تُظهر الملصقات الملونة نوع الخدمة ومكانها. ويجب أن تشير ملصقات تدوين الملاحظات تحتها إلى الأشخاص الذين تستهدفهم هذه الخدمات والأشخاص الذين يتمتعون بفرصة أكبر بالوصول إليها.

ب.2 ما هي الطريقة التي يمكن من خلالها للـ[مرأة في القصة أعلاه] وغيرها من النساء الحصول على المعلومات حول الخدمات المتوفرة؟ هل هي موفرة بسهولة؟

ب.2 أ هل تعتقد أن طرقك في نشر المعلومات حول الخدمات فعالة في الوصول إلى النساء؟ والناجيات من العنف القائم على نوع الجنس؟

ب.2 أ 1 إذا كان الجواب نعم، كيف تنشر هذه المعلومات بين مجموعات مستهدفة مختلفة (رجال، نساء، مراهقون، ذوو إعاقات، أشخاص لديهم احتياجات محددة في مجال الصحة النفسية)؟

ب2أ2 إذا كان الجواب لا، ما الذي يمكن فعله لتحسين ذلك؟

ب.3 هل تعتقد أن النساء يشعرن بالارتياح عند السعي للحصول على هذه الخدمات؟ والناجيات من العنف القائم على نوع الجنس؟ وما هو السبب؟

ب.4 ما نوع الحواجز التي تمنع النساء بشكل عام من الوصول إلى هذه الخدمات؟ (احتمالات: الحواجز الجغرافية والأمنية، الحواجز العائلية/الاجتماعية، إلخ.). أثناء إجابة المشاركين على السؤالين ب.4 وب.4 أ، املأ الجدول أدناه، ثم اسأل عن أهم 3 حواجز، مع تمييزها/تسطيرها بلون مختلف.

ب.4 أ ما الذي يمكن القيام به لتحسين ذلك؟

الجدول 1.4: لائحة الحواجز التي تمنع الناجيات من العنف القائم على نوع الجنس من الوصول إلى الخدمات مع اعتبارات في مجال الصحة النفسية	الحلول المحتملة
1.2.1	
1.2.2	
1.2.3	
1.2.4	
1.2.5	
1.2.6	
1.2.7	
1.2.8	

	1.2.9
	1.2.10

القسم ج. أسئلة محددة حول الناجيات من العنف القائم على نوع الجنس واللواتي لديهن احتياجات محددة في مجال الصحة النفسية

ج.1 ما هي المشاكل التي تشكل أكبر مصدر للقلق بالنسبة للناجيات من العنف القائم على نوع الجنس واللواتي لديهن احتياجات محددة في مجال الصحة النفسية في [الموقع]؟ يرجى إدرج أكبر عدد من المشاكل التي يمكنك التفكير فيها. الميسر: اكتب هذه المشاكل في هذا الجدول. قد يشمل العنف القائم على نوع الجنس العنف الجسدي (مثل الضرب والدفع والطعن وغيرها من الأعمال الضارة التي تؤذي المرأة جسدياً) والعنف العاطفي/النفسي (مثل الإذلال وحرمان المرأة من رؤية عائلتها/أصدقائها وتخويفها وعزلها) والعنف الجنسي (أداء أفعال جنسية من أي نوع ضد إرادة الشخص أو دون موافقته)، و/أو العنف الاقتصادي (على سبيل المثال منع المرأة من الوصول إلى الموارد النقدية أو اتخاذ أي قرارات تتعلق بالمال، وعدم السماح للنساء بالحصول على المال لتلبية الاحتياجات الأساسية مثل الماء أو الطعام، أو الاحتياجات الشخصية). قد تشمل الاحتياجات في مجال الصحة النفسية أعراض الاكتئاب والقلق والاضطرابات الذهانية/العصبية، والإجهاد، والإجهاد الضار، وغيرها من الأعراض التي قد تحتاج المرأة التي تعاني منها إلى دعم أو تدخل.

المشكلة	الوصف
1.1.1	
1.1.2	
1.1.3	
1.1.4	
1.1.5	
1.1.6	
1.1.7	
1.1.8	
1.1.9	
1.1.10	
1.1.11	
1.1.12	
1.1.13	
1.1.14	
1.1.15	
1.1.16	
1.1.17	
1.1.18	
1.1.19	
1.1.20	

ج.1 أ هل تختلف هذه المشاكل عن المشاكل التي تواجهها نساء أُخريات؟

ج.1 ب إذا كانت تختلف، فكيف؟ وهل هناك حاجة إلى تقديم خدمات محددة لهذه المجموعة المستهدفة؟ (إذا كان الأمر كذلك، فكر في نوع الخدمات، وتوافرها).

ج.2 الميسِّر: بناءً على ما سبق، أعطِ مثالاً عن امرأة قد تكون تعاني من المشاكل المذكورة أعلاه. أعطِ هذه المرأة اسماً، وعمراً، وحدد نوع المنطقة التي تقيم فيها منزلها، ثم أدرج المشاكل التي قد تكون تواجهها (استناداً إلى الأمثلة المذكورة أعلاه). ثم اسأل المشاركين: ما هي الحواجز التي قد تمنع هذه المرأة بشكل خاص، وغيرها من النساء الناجيات من العنف القائم على نوع الجنس واللواتي لديهن احتياجات في مجال الصحة النفسية، من الوصول إلى الخدمات التي تحدثنا عنها؟

مثال: لمى (37 عاماً، من لبنان، تعيش في منطقة ريفية)، أخبرتك سراً بأنها تعاني من نوبات هلع كل أسبوع ولا تستطيع مغادرة السرير معظم الأيام. أخبرتك أن والدها أصبح رجلاً غاضباً، وأنه يدفعها ويهينها أمام عائلتها لأنها غير متزوجة. تشعر لمى بعدم الأمان عند السير في الشوارع وحدها لأنها تعرضت لاعتداء جنسي ومضايقة من جيرانها، ولأنها تتعرض للسخرية بسبب إصابتها بمتلازمة داون. ما هي الخدمات التي توصي بها لها في المنطقة؟ أمثلة أخرى عن المشاكل التي قد تطرأ على بعض النساء: عدم الوصول إلى الدعم النفسي والاجتماعي لمعالجة حالات الضيق، والمشاكل الزوجية التي تنتهي بالعنف، وعدم الوصول إلى المال لتلبية الاحتياجات الأساسية أو الشخصية، وعدم القدرة على الذهاب لرؤية الأصدقاء/السعي للحصول على الدعم النفسي والاجتماعي بسبب العنف الذي يمارسه الشريك، إلخ.

ج.2 أ ما هي بعض طرق العمل للتغلب على هذه العقبات؟

ج.2 ب هل تعرف آلية يتم استخدامها في منطقتك للقيام بذلك؟

ج.2.ج ما الذي يمكن أن يفعله قادة المجتمع/مقدمو الخدمات على وجه التحديد لتحقيق ذلك؟

اختتم المناقشة[4]

[4] لجنة الإنقاذ الدولية (2011). مجموعة أدوات التقييم الخاصة بلجنة الإنقاذ الدولية.

- أشكر المشاركين على وقتهم ومساهماتهم.
- ذكّر المشاركين بأن الغرض من هذه المناقشة هو فهم احتياجات وشواغل النساء والفتيات بشكل أفضل منذ الأزمة.
- ذكّر المشاركين بموافقتهم على السرية.
- ذكّر المشاركين بعدم مشاركة المعلومات أو أسماء المشاركين الآخرين مع أفراد آخرين في المجتمع.
- اسأل المشاركين إذا كان لديهم أسئلة.
- إذا رغب أحد في التحدث على انفراد، فأجب بأن الميسر وأمين السر سيكونان متاحين بعد الاجتماع.
