# Supplementary material for: “I Go up to the Edge of the Valley, and I Talk to God”: Using Mixed Methods to Understand the Relationship between Gender-Based Violence and Mental Health among Lebanese and Syrian Refugee Women Engaged in Psychosocial Programming
Source: Int J Environ Res Public Health. 2021 Apr 23;18(9):4500. doi: 10.3390/ijerph18094500 (PMC8123009; doi:10.3390/ijerph18094500)
Supplement: Supplementary file 1 [file ijerph-18-04500-s001.zip › Quantitative Tool/Abaad GWI Quantitative Survey - ENG_ARA .docx.pdf]

أبعاد
abaad

The Global Women's Institute
THE GEORGE WASHINGTON UNIVERSITY

ADMINISTRATION FORM

IDENTIFICATION		
LOCATION OF INTERVIEW<br>موقع المقابلة		BEBNINE.………………..1<br>QOBBEH……..…………..2<br>BAALBAK ……….…..….3<br>LABWE ……………..……4<br>OTHER ………...….…..…….5<br>(Please specifiy)
INTERVIEWER CONTACT		
INTERVIEWER NAME OR CODE<br>اسم الشخص الذي يجري المقابلة أو رمزه		CODE[ ]
FIELD SUPERVISOR<br>**المشرف الميداني--نقطة الاتصال**		SELMA …….……………1<br>TONINA ………………...2
QUESTIONNAIRES COMPLETED?	REFUSED ……………………………………………..1<br>(specify reason) ______________________________<br><br>COULD NOT BE CONTACTED ………………...……2<br>POSTPONED INTERVIEW ……………………...……3<br>INCAPACITATED ……...……………………..……...4<br>PARTIALLY COMPLETED …...………………...…….5<br>COMPLETED ……………...………………………….**6**	□**RECONTACT**<br><br>□**RECONTACT**

INTRODUCTION AND CONSENT

Hello, my name is ______________________ . I am interviewing you on behalf of ABAAD and the Global Women's Institute. We are conducting a survey among displaced and host communities in Lebanon, to learn about women's health and well-being.

Read the following informed consent text out loud to the interviewee:
Title of research study: "Relationship between GBV and Mental Health among Female Survivors in Lebanon"

Researchers: Eugene Sensenig (Principal Investigator) from FLPS NDU, Saja Michael (Lead Researcher) and Rassil Barada (Field Research Coordinator) from ABAAD (Resource Center for Gender Equality), in partnership with Alina Potts at the Global Women's Institute at George Washington University (Washington DC, USA).

Why am I being invited to take part in a research study? We invite you to take part in a research study because we hope it will contribute to our understanding of how to better support women survivors of GBV with specific mental health needs.

What should I know about a research study?

- *Someone will explain this research study to you.*
- *Whether or not you take part is up to you.*
- *You can choose not to take part.*
- *You can agree to take part and later change your mind.*
- *Your decision will not be held against you.*
- *You can ask all the questions you want before you decide.*

Who can I talk to? If you have questions, concerns, or complaints, or think the research has hurt you, talk to the research team. The lead researcher for this study is Saja Michael, and the field research coordinator is Rassil Barada. You can contact either of them, or Alina Potts from the Global Women's Institute with the below information. ABAAD Furn El Chebbak, Sector 5, 51 Bustani Street, Najjar Bldg., Ground Floor Beirut, Lebanon abaad@abaadmena.org +961 1 283 820 You can also contact Alina Potts, Global Women's Institute, George Washington University, email: apotts@email.gwu.edu As well as the Primary Investigator from Notre Dame University, Louaize, Lebanon, Dr. Eugene Sensenig, at esensenig@ndu.edu.lb This research has been reviewed and approved by an Institutional Review Board ("IRB"). You may talk to them at [fill in once completed].

- *Your questions, concerns, or complaints are not being answered by the research team.*
- *You cannot reach the research team.*
- *You want to talk to someone besides the research team. 🎬 You have questions about your rights as a research subject.*
- *You want to get information or provide input about this research.*

Why is this research being done? We hope this research will contribute to our understanding of how to better support women survivors of GBV with specific mental health needs. We also hope to get more thorough information on the barriers that these women face in accessing any necessary services.

<u>How long will the research last?</u> If you agree to participate, you may be asked to participate in an individual interview with me now, for approximately [60] minutes. Time will be spent answering questions with a social worker.

<u>How many people will be studied?</u> We expect about 1000 people will participate in this study.

<u>What happens if I say yes, I want to be in this research?</u> Participation will involve answering questions about a variety of topics including your exposure to various forms of gender-based violence, experiences of mental health symptoms, and some coping methods.

<u>What happens if I do not want to be in this research?</u> You can leave the research at any time and it will not be held against you.

<u>Risks</u>
There are some risks to participating.

- *The issues we are discussing can be sensitive and upsetting. You are welcome to skip any questions you do not feel comfortable answering. If at any point, you feel uncomfortable or upset, you are welcome to excuse yourself.*
- *The topics we are discussing may be controversial and others may express opinions different from your own. In the event that a dispute arises, the facilitator will redirect the conversation and remind participants to remain respectful of others' opinions.*
- *All participants in this study are encouraged to call ABAAD if the study causes any discomfort or distress, and are all already beneficiaries of ABAAD and have trusted relationship with the organization. If there is any discomfort or distress, please let us know if you would like to take a break or stop the interview.*
- *If you would like to speak to someone following the discussion about accessing services, please contact ABAAD representative Miss Saja Michael [local Lebanese phone number] or Rassil Barada [local Lebanese phone number].*
- *If you would like to speak to someone about your rights as a research participant, please contact ABAAD representative Miss Saja Michael [local Lebanese phone number], Ms. Rassil Barada[local Lebanese phone number], Alina Potts [apotts@email.gwu.edu]*

<u>Benefits</u>
There are also benefits to participation.

- *This is an opportunity to hear others' opinions and express yourself in a safe space*
- *Your perspective will be valued highly by our research team*
- *Your contributions can assist in developing future programs for women survivors of GBV with specific mental health considerations*

By consenting to the above, you are indicating that you understand the above information and agree to take part in this study. Please understand that you may withdraw your consent at any time without penalty, and that, by giving consent, you are not waiving any legal claims or rights. Before proceeding, you can ask any questions about what it means to give informed consent.

NOTE TO RESEARCHER: OBTAIN CONSENT BY CLICKING OK ON TABLET. IF PERSON DOES NOT CONSENT, THANK THEM AND END INTERACTION.

مرحباً، اسمي ______________. أنا أنا أقوم بإجراء المقابلة معك لدراسة مع أبعاد والمعهد العالمي للمرأة. نحن نجري إستمارة معلومات بين المجتمعات النازحة والمستضيفة في لبنان للحصول على المزيد من المعلومات حول صحة ورفاه النساء

[يرجى قراءة بيان الموافقة أدناه]

عنوان الدراسة البحثية: العلاقة بين العنف القائم على النوع الإجتماعي والصحة النفسية بين الناجيات في لبنان

لماذا دُعيت للمشاركة في دراسة بحثية؟

ندعوكِ للمشاركة في دراسة بحثية لأننا نأمل أن يساهم ذلك في فهمنا لكيفية تقديم الدعم بشكل أفضل للسيدات

ما الذي يجب أن أعرفه عن الدراسة البحثية؟

- سوف يشرح لكِ شخص ما هذه الدراسة البحثية.
- الأمر متروك لكِ بالمشاركة أم لا.
- يمكنك اختيار عدم المشاركة.
- يمكنكِ الموافقة على المشاركة وتغيير رأيك في ما بعد.
- قرارك لن يُستخدم ضدك.
- يمكنكِ طرح كل الأسئلة التي تريدينها قبل اتخاذ القرار.

مع مَن يمكنني أن أتحدث؟ إذا كانت لديك أسئلة أو مخاوف أو شكاوى أو إذا كنت تعتقدين أن البحث أضرَّ بكِ، تحدثي إلى فريق البحث. الباحثة الرئيسية في هذه الدراسة هي سجى مخايل، ومنسقة البحوث الميدانية هي راسيل بردة. يمكنكِ الاتصال بإحداهما، أو بألينا بوتس من المعهد العالمي للمرأة وفق المعلومات أدناه.

abaad@abaadmena.org
+961 1 283 820
81 78 81 78 (safe line)

[لقد تمت مراجعة هذا البحث والموافقة عليه من قبل لجنة مؤسسية. يمكنكِ التحدث إليهم على [يُملأ عند الانتهاء.

- لم يُجِب فريق البحث على أسئلتك أو مخاوفك أو شكاويكِ.
- لا يمكنكِ الوصول إلى فريق البحث.
- تريدين التحدث إلى شخص ما بالإضافة إلى فريق البحث.
- لديكِ أسئلة عن حقوقك كموضوع بحثي.
- تريدين الحصول على معلومات أو تقديم مدخلات حول هذا البحث.

لماذا يجرى هذا البحث؟

نأمل أن يسهم هذا البحث في فهمنا لكيفية تقديم الدعم بشكل أفضل للنساء الناجيات من العنف القائم على نوع الجنس اللواتي لديهن احتياجات خاصة في مجال الصحة النفسية. كما نأمل في الحصول على معلومات أكثر شمولاً عن الحواجز التي تواجه هؤلاء النساء في الوصول إلى أي خدمات ضرورية.

إذا وافقتِ على المشاركة، قد يُطلب منك المشاركة في مقابلة فردية معي الآن لمدة [45 دقيقة] تقريباً

ما عدد الأشخاص الذين سيشاركون في الدراسة؟ نتوقع أن يشارك حوالي 1000 شخص في هذه الدراسة.

ما الذي سيحدث إذا قلت نعم، أريد المشاركة في هذا البحث؟

ستشمل المشاركة الإجابة عن أسئلة حول مجموعة متنوعة من الموضوعات بما في ذلك تعرضك لأشكال مختلفة من العنف القائم على النوع الإجتماعي، و الصحة النفسية، وبعض طرق التكيف.

ما الذي سيحدث إذا لم أوافق على المشاركة في هذا البحث؟ يمكنك التوقف عن المشاركة في البحث في أي وقت ولن يُستخدم قرارك ضدك.

- قد تكون القضايا التي نناقشها حساسة ومقلقة. يمكنك اختيار عدم الإجابة على أسئلة لا تشعرين بالراحة في الإجابة عليها. إذا شعرتِ بعدم الارتياح أو الانزعاج في أي وقت، يمكنك الانسحاب.
- بإمكانك الاتصال بأبعاد إذا كانت الدراسة تسبب أي إزعاج أو ضائقة. إذا كان هنالك أي إزعاج أو ضائقة، يرجى إعلامنا إذا كنت ترغبين في أخذ استراحة أو إيقاف المقابلة.
- إذا كنت ترغبين في التحدث إلى شخص ما بعد المناقشة حول الوصول إلى الخدمات، يرجى التواصل مع احد اعضاء أبعاد في المركز أو من خلال الخط الساخن

الفوائد: للمشاركة فوائد كذلك.

- إنها فرصة لسماع آراء الآخرين والتعبير عن نفسك في مكان آمن.
- سيقدِّر فريق بحثنا وجهة نظرك بدرجة عالية.
- يمكن لمساهماتك أن تساعد في تطوير برامج مستقبلية للنساء الناجيات من العنف القائم على نوع الجنس مع واللواتي لديهم احتياجات خاصة في مجال الصحة النفسية.

بالموافقة على ما ورد أعلاه، فإنك تشيرين إلى أنك تفهمين المعلومات المذكورة أعلاه وتوافقين على المشاركة في هذه الدراسة.
يرجى الملاحظة أنه يمكنك سحب موافقتك في أي وقت بدون عقوبة، وأنك من خلال الموافقة، لا تتنازلين عن أي مطالبات قانونية أو حقوق.

قبل المتابعة، يمكنك طرح أي أسئلة حول معنى منح الموافقة المستنيرة.

Consent to be interviewed?<br>هل توافق المستفيدة على إجراء المقابلة؟	AGREES TO BE INTERVIEWED ……………….**1**<br>**وافقت على إجراء المقابلة**<br><br>DOES NOT AGREE TO BE INTERVIEWED…....**2**<br>**لم توافق على إجراء المقابلة**	GO TO QUESTION 1<br>انتقل إلى السؤال 1<br><br>THANK HER FOR HER TIME AND CONSIDERATION, END INTERACTION<br>شكرا لها على وقتها والنظر ، والتفاعل نهاية

	**DEMOGRAPHIC INFORMATION**<br>**المعلومات الديمغرافية**		
Q1	I will start by asking you a few general questions.<br><br>How old are you?<br>سأبدأ بطرح بعض الأسئلة العامة. كم عمرك؟<br><br>(IN COMPLETED YEARS)<br>بالأعوام المكتملة	AGE (YEARS) [ ]<br>(العمر (سنوات)	
Q2	What is your nationality?<br>ما هي جنسيتك؟	LEBANESE (1 ……………….………(لبنانية<br>SYRIAN (2 ..………..……..……….(سوري<br>PALESTINIAN (3 …………....…(فلسطينية<br><br>OTHER, please specify (:(غير ذلك<br>______________________ 4<br>DON'T KNOW/DON'T REMEMBER 98<br>REFUSED/NO ANSWER 99	□SKIP TO Q2b<br>انتقل إلى السؤال 2 ب
Q2b	How long have you been living in Lebanon?<br>منذ متى تعيشين في لبنان؟	LESS THAN 1 YEAR (1..(أقل من عام واحد<br>1 - 2 YEARS (2……………(عام واحد-عامان<br>3 - 5 YEARS (5-3 (أعوام 3<br>5 - 7 YEARS (7-5 (أعوام 4<br>OVER 7 YEARS (> 7 (أعوام 5<br>DK/DOES NOT WISH TO DISCLOSE<br>((لا أعرف/لا أذكر 9	
Q2c	Did you migrate to Lebanon because of the war in Syria?<br>هل هاجرت إلى لبنان بسبب الحرب في سوريا؟	YES (1………………...………………(نعم<br>NO (2……………………………….(لا	
Q3	What is your current marital status?<br>ما هو وضعك الاجتماعي الحالي؟	MARRIED, LIVING WITH SPOUSE<br>متزوجة/أعيش مع شريك...1<br>MARRIED, NOT LIVING W/SPOUSE<br>متزوجة، لا أعيش مع شريك....2<br>ENGAGED TO BE MARRIED<br>مخطوبة، سوف أتزوج....3<br>DIVORCED OR SEPARATED<br>كنت متزوجة-- مطلقة أو منفصلة عن زوجي....4<br>WIDOWED<br>أرملة....5<br>SINGLE<br>عزباء....6<br>OTHER (PLEASE SPECIFY)<br>(غير ذلك)....7	IF 1,2,3,4,5<br>□SKIP TO Q3c<br><br>إذا كان 1،2،3،4،5 انتقل إلى السؤال 3 ج

Q3b	**[IF SINGLE OR OTHER IN Q5]**<br><br>Have you ever been in a relationship with a male partner?<br>هل كنت يوماً على علاقة دائمة بشريك؟<br><br>Prompt: This includes relationships where you lived together or lived separately.<br>[قد يشمل ذلك الشركاء الذين يقيمون معاً أو بشكل منفصل]	YES<br>نعم......1<br>NO<br>لا.......2<br>DOESN'T KNOW/REMEMBER<br>لا أعرف / تذكر.......8<br>DOES NOT WISH TO DISCLOSE<br>لا ترغب في الكشف عنها 9	□SKIP TO Q4<br>انتقل إلى السؤال 4
**IF EVER MARRIED [Q7-10]**			
Q3c	At what age did you first get married?<br>في أي عمر تزوجتي؟<br>(If unable to answer, what year did you first get married?)<br>إن لم تتمكن من الإجابة عن العمر: في أي سنة تزوجت للمرة الأولى؟	AGE (YEARS) [ ]<br>(العمر (سنوات<br><br>LAST 2 DIGITS OF MARRIAGE YEAR [ ]<br>سنة الزواج	
Q3d	[IF EVER MARRIED]<br><br>Did you choose your husband or did someone else choose him for you?<br>هل اخترت زوجك الراهن أو الأخير بنفسك أو اختاره لك شخص آخر؟<br>REFER TO CURRENT OR MOST RECENT HUSBAND<br><br>IF SHE DID NOT CHOOSE HERSELF, PROBE: Who chose your current/most recent husband for you?<br>أشِر إلى الزوج الراهن أو الأخير<br>إذا لم تكن قد اختارته بنفسها، اسأل: من اختار لك زوجك الراهن/الأخير؟	BOTH CHOSE<br>اختاره الطرفان معاً .... 1<br>SHE CHOSE<br>اختارته المستجيبة ....2<br>HER FAMILY CHOSE<br>اختارته عائلة المستجيبة....3<br>PARTNER CHOSE<br>الشريك هو من اختار ....4<br>HIS FAMILY CHOSE<br>عائلة الشريك هي من اختار ....5<br>DOESN'T KNOW/REMEMBER<br>لا أعرف / تذكر.......8<br>DOES NOT WISH TO DISCLOSE<br>لا ترغب في الكشف عنها 9	□SKIP TO 3g<br>انتقل إلى السؤال 3 ز<br><br>□SKIP TO 3g<br>انتقل إلى السؤال 3 ز
Q3f	Did [the person who chose your husband] ask you whether you wanted to marry him or not?<br>هل سألت إذا كنت تريدين الزواج منه أم لا؟<br>REFER TO CURRENT OR MOST RECENT HUSBAND<br>أشِر إلى الزوج الراهن أو الأخير	YES<br>نعم......1<br>NO<br>لا.......2<br>DOESN'T KNOW/REMEMBER<br>لا أعرف / تذكر.......8<br>DOES NOT WISH TO DISCLOSE<br>لا ترغب في الكشف عنها 9	
Q3g	How old is your [current] husband?<br>كم عمر زوجك [الراهن]؟<br><br>(IN COMPLETED YEARS)	AGE (YEARS) [ ]<br>(العمر (سنوات	

ALL WOMEN [Q11-14]			
Q4	Have you ever been pregnant?<br>هل سبق لك الحمل؟	YES<br>نعم......1<br>NO<br>لا......2<br>DOESN'T KNOW/REMEMBER<br>لا أعرف / تذكر......8<br>DOES NOT WISH TO DISCLOSE<br>لا ترغب في الكشف عنها 9	SKIP TO Q5<br>انتقل إلى السؤال 5
Q4a	Have you had children?<br>هل رُزقت بأطفال؟	YES<br>نعم......1<br>NO<br>لا......2<br>DOESN'T KNOW/REMEMBER<br>لا أعرف / تذكر......8<br>DOES NOT WISH TO DISCLOSE<br>لا ترغب في الكشف عنها 9	SKIP TO Q4c<br>انتقل إلى السؤال 4 ج
Q4b	How many children do you have?<br>كم طفلاً لديك؟	NONE لا أطفال 1<br>1 – 2 طفل واحد-طفلان .....2<br>3 – 5 أطفال 3-5 3<br>MORE THAN 5 أكثر من 5 أطفال 4	
Q4c	How old were you when you first became pregnant?<br>كم كان عمرك عندما حملت للمرة الأولى؟<br>إذا قالت لا ، اكتب 99<br><br>(IN COMPLETED YEARS)	AGE (YEARS) [ ]<br>العمر (سنوات)	
Q5	What is the highest level of education that you achieved? MARK HIGHEST LEVEL.<br>ما هو أعلى مستوى علمي بلغته؟<br><br>*(Primary: grades 1-6; Secondary: grades 7-12. Higher is vocational/technical schools/University/etc)*<br>اذكر أعلى مستوى تم بلوغه<br>*ابتدائي: الصف 1-8؛ ثانوي: الصف 9-12. التعليم العالي [أي المدارس المهنية/التقنية/الجامعة/إلخ]"*	NONE<br>لا شيء ...1<br>PRIMARY (SOME)<br>ابتدائي (غير متمم) ...2<br>PRIMARY (COMPLETED)<br>ابتدائي (متمم) ...3<br>SECONDARY (SOME)<br>ثانوي (غير متمم) ...4<br>SECONDARY (COMPLETED)<br>ثانوي (متمم) ...5<br>HIGHER (SOME)<br>عالي (غير متمم) ...6<br>HIGHER (COMPLETED)<br>عالي (متمم) ...7<br><br>DOESN'T KNOW/REMEMBER<br>لا أعرف / تذكر......8<br>DOES NOT WISH TO DISCLOSE	

		لا ترغب في الكشف عنها<br>9........	
Q6	**[IF EVER MARRIED]**<br><br>What is the highest level of education that your husband has achieved?<br>ما هو أعلى مستوى علمي بلغه زوجك؟<br>اذكر أعلى مستوى تم بلوغه<br><br>*(Primary: grades 1-6; Secondary: grades 7-12. Higher is vocational/technical schools/University/etc)*<br>اذكر أعلى مستوى تم بلوغه<br>*ابتدائي: الصف 1-8؛ ثانوي: الصف 9-12. التعليم العالي [أي المدارس المهنية/التقنية/الجامعة/إلخ"]*	NONE<br>لا شيء ...1<br>PRIMARY (SOME)<br>ابتدائي (غير متمم) ...2<br>PRIMARY (COMPLETED)<br>ابتدائي (متمم) ...3<br>SECONDARY (SOME)<br>ثانوي (غير متمم) …4<br>SECONDARY (COMPLETED)<br>ثانوي (متمم) …5<br>HIGHER (SOME)<br>عالي (غير متمم) …6<br>HIGHER (COMPLETED)<br>عالي (متمم) …7<br><br>DOESN'T KNOW/REMEMBER<br>لا أعرف / تذكر…….8<br>DOES NOT WISH TO DISCLOSE<br>لا ترغب في الكشف عنها ……..9	
**HOUSEHOLD SOCIOECONOMIC STATUS**<br>**الوضع الاجتماعي والاقتصادي للأسرة**			
Q7	Are you employed?<br>هل تعملين؟	YES<br>نعم……1<br>NO<br>لا…….2<br>DOESN'T KNOW/REMEMBER<br>لا أعرف / تذكر…….8<br>DOES NOT WISH TO DISCLOSE<br>لا ترغب في الكشف عنها ……..9	□**SKIP TO Q9**<br>**انتقل إلى السؤال 9**
Q8	What do you do mainly for work?<br>ما هو عملك؟<br><br>(إذا غير ذلك (يرجى التحديد	AGRICULTURE<br>في الزراعة …1<br>SMALL BUSINESS<br>مشروع صغير …2<br>DAILY LABORER<br>عاملة يومية …3<br>CONTRUCTION<br>في البناء…4<br>DOMESTIC WORK<br>عمل منزلي…5<br>MILITARY<br>في الجيش…6<br>GOVERNMENT<br>موظفة حكومية …7<br>RETIRED	

		متقاعدة ...8<br>OTHER (Please specify)<br>المساعدة/الاحتياجات الأساسية ...9<br><br>DOESN'T KNOW/REMEMBER<br>لا أعرف / تذكر...98	
Q9	**[IF MARRIED]**<br>Is your husband employed?<br>هل زوجك موظف؟	YES<br>نعم......1<br>NO<br>لا.......2<br>DOESN'T KNOW/REMEMBER<br>لا أعرف / تذكر.......8<br>DOES NOT WISH TO DISCLOSE<br>لا ترغب في الكشف عنها 9	SKIP TO Q11<br>انتقل إلى السؤال 11
Q10	What does he mainly do for work?<br>ما هو عمله؟	AGRICULTURE<br>في الزراعة ...1<br>SMALL BUSINESS<br>مشروع صغير ...2<br>DAILY LABORER<br>عاملة يومية ...3<br>CONTRUCTION<br>في البناء...4<br>DOMESTIC WORK<br>عمل منزلي...5<br>MILITARY<br>في الجيش...6<br>GOVERNMENT<br>موظفة حكومية ...7<br>RETIRED<br>متقاعدة ...8<br>OTHER (Please specify)<br>المساعدة/الاحتياجات الأساسية ...9<br><br>DOESN'T KNOW/REMEMBER<br>لا أعرف / تذكر...98	
Q11	What is the main source of income for you and your household?<br>ما هو المصدر الأساسي للمال بالنسبة لك ولأسرتك؟	NO INCOME<br>ما من مصدر دخل...1<br>MONEY FROM YOUR OWN WORK<br>مال من عملي الخاص ...2<br>SUPPORT FROM HUSBAND<br>دعم من الزوج/الشريك ...3<br>SUPPORT FROM PARENTS<br>دعم من الأهل ...4<br>SOCIAL SERVICES<br>الخدمات الاجتماعي ...5<br>HUMANITARIAN AID<br>المساعدات الإنسانية ...7<br>SUPPORT FROM OTHER RELATIVES<br>دعم من أقارب آخرين ...8	

		OTHER (Please specify)<br>غير ذلك...9	
**ENVIRONMENTAL VULNERABILITIES<br>(taken from the HESPER scale)<br>(نقاط الضعف البيئية (مأخوذة من مقياس الاحتياجات الملحوظة في أطر حالات الطوارئ الإنسانية**			
Q12	Do you have a serious problem with food?<br>PROBE: For example, because you do not have enough food, or good enough food, or because you are not able to cook food<br>هل تعانين من مشكلة معينة متعلقة بالطعام؟ مثلاً لأنك لا تملكين ما يكفي من الطعام أو طعاماً جيداً بما يكفي أو لأنك لا تستطيعين طهو الطعام	NO SERIOUS PROBLEM<br>ما من صعوبات ...0<br>SERIOUS PROBLEM<br>صعوبات ...1<br>DK/DOES NOT WISH TO DISCLOSE<br>لا أعرف/لا أذكر ...9	
Q13	Do you have a serious problem because you do not have easy and safe access to a clean toilet?<br>هل تواجهين صعوبات لأنك لا تستطيعين الوصول بسهولة وبأمان إلى مرحاض نظيف؟	NO SERIOUS PROBLEM<br>ما من صعوبات ...0<br>SERIOUS PROBLEM<br>صعوبات ...1<br>DK/DOES NOT WISH TO DISCLOSE<br>لا أعرف/لا أذكر ...9	
Q14	Do you have a serious problem with your physical health?<br>PROBE: For example, because you have a physical illness, injury or disability?<br>هل تعانين من صعوبات متعلقة بصحتك الجسدية؟ مثلاً، هل تعانين من مرض أو إصابة أو إعاقة جسدية	NO SERIOUS PROBLEM<br>ما من صعوبات ...0<br>SERIOUS PROBLEM<br>صعوبات ...1<br>DK/DOES NOT WISH TO DISCLOSE<br>لا أعرف/لا أذكر ...9	
Q15	Do you have a serious problem because you or your family are not safe or protected where you live now?<br>PROBE: For example, because of conflict, violence or crime in your community, city, village, or household.<br>هل تعانين من صعوبات لأن عائلتك ليست آمنة أو محمية في المكان الذي تعيش فيه اليوم؟ مثلاً بسبب الصراع أو العنف أو الجرائم في مجتمعك أو مدينتك أو قريتك أو منزلك؟	NO SERIOUS PROBLEM<br>ما من صعوبات ...0<br>SERIOUS PROBLEM<br>صعوبات ...1<br>DK/DOES NOT WISH TO DISCLOSE<br>لا أعرف/لا أذكر ...9	
Q16	Do you have a serious problem because you are separated from family members?<br>PROBE: For example, because of conflict, violence, or displacement.<br>هل تعانين من صعوبات لأنك منفصلة عن أفراد عائلتك؟ مثلاً بسبب الصراع أو العنف أو النزوح	NO SERIOUS PROBLEM<br>ما من صعوبات ...0<br>SERIOUS PROBLEM<br>صعوبات ...1<br>DK/DOES NOT WISH TO DISCLOSE<br>لا أعرف/لا أذكر ...9	

**MENTAL AND PHYSICAL HEALTH**<br>**الذي ينفذه مجري المقابلة K6 الاختبار بمقياس**			
**READ ALOUD:** The next questions are about how you have been feeling during the past 30 days.<br>قراءة ما يلي بصوت عال<br>تتعلق الأسئلة التالية بشعورك في الـ30 يوماً الاخيرة. أود أن أذكرك بأن جميع المعلومات الواردة في هذا الاستبيان سرية ولن تتم مشاركتها مع أي شخص.			
Q17	About how often during the past 30 days did you feel <u>nervous</u> — would you say all of the time, most of the time, some of the time, a little of the time, or none of the time?<br>بمدى شعورك بالعصبية في الـ *30* يوماً الأخيرة- هل تعتبرين أنك شعرت بالعصبية طوال الوقت أو في غالبية الوقت أو لبعض الوقت أو لقليل من الوقت أو أبداً؟	ALL<br>طوال الوقت ...1<br>MOST<br>في غالبية الوقت ...2<br>SOME<br>لبعض الوقت ...3<br>A LITTLE<br>لقليل من الوقت ...4<br>NONE<br>أبداً ...5<br><br>DOESN'T KNOW/REMEMBER<br>لا أعرف / تذكر...8<br>DOES NOT WISH TO DISCLOSE<br>لا ترغب في الكشف عنها ...9	
Q18	During the past 30 days, about how often did you feel <u>hopeless</u> — would you say all of the time, most of the time, some of the time, a little of the time, or none of the time?<br>في الأيام الـ *30* الأخيرة، ما كان مدى شعورك باليأس- طوال الوقت أو في غالبية الوقت أو لبعض الوقت أو لقليل من الوقت أو أبداً؟-	ALL<br>طوال الوقت ...1<br>MOST<br>في غالبية الوقت ...2<br>SOME<br>لبعض الوقت ...3<br>A LITTLE<br>لقليل من الوقت ...4<br>NONE<br>أبداً ...5<br><br>DOESN'T KNOW/REMEMBER<br>لا أعرف / تذكر...8<br>DOES NOT WISH TO DISCLOSE<br>لا ترغب في الكشف عنها ...9	

Q19	During the past 30 days, about how often did you feel <u>restless or fidgety</u>?<br><br>*IF NECESSARY: all, most, some, a little, or none of the time?*<br>في الأيام الـ 30 الأخيرة، ما كان مدى شعورك بالقلق أو التوتر؟<br>(عند الضرورة: طوال الوقت أو في غالبية الوقت أو لبعض الوقت أو لقليل من الوقت أو أبداً؟)	ALL<br>طوال الوقت ...1<br>MOST<br>في غالبية الوقت ...2<br>SOME<br>لبعض الوقت ...3<br>A LITTLE<br>لقليل من الوقت ...4<br>NONE<br>أبداً ...5<br><br>DOESN'T KNOW/REMEMBER<br>لا أعرف / تذكر...8<br>DOES NOT WISH TO DISCLOSE<br>لا ترغب في الكشف عنها ...9	
Q20	How often did you feel so <u>depressed</u> that nothing could cheer you up?<br><br>*IF NECESSARY: all, most, some, a little, or none of the time?*<br>ما كان مدى شعورك بالاكتئاب إلى حد أن لا شيء استطاع إسعادك؟<br>(عند الضرورة: طوال الوقت أو في غالبية الوقت أو لبعض الوقت أو لقليل من الوقت أو أبداً؟)	ALL<br>طوال الوقت ...1<br>MOST<br>في غالبية الوقت ...2<br>SOME<br>لبعض الوقت ...3<br>A LITTLE<br>لقليل من الوقت ...4<br>NONE<br>أبداً ...5<br><br>DOESN'T KNOW/REMEMBER<br>لا أعرف / تذكر...8<br>DOES NOT WISH TO DISCLOSE<br>لا ترغب في الكشف عنها ...9	
Q21	During the past 30 days, about how often did you feel that everything was an effort?<br><br>*IF NECESSARY: all, most, some, a little, or none of the time?*<br>في الأيام الـ 30 الأخيرة، ما كان مدى شعورك بأنك تحتاجين لبذل الكثير من الجهد للقيام بالأمور؟<br>(عند الضرورة: طوال الوقت أو في غالبية الوقت أو لبعض الوقت أو لقليل من الوقت أو أبداً؟)	ALL<br>طوال الوقت ...1<br>MOST<br>في غالبية الوقت ...2<br>SOME<br>لبعض الوقت ...3<br>A LITTLE<br>لقليل من الوقت ...4<br>NONE<br>أبداً ...5<br><br>DOESN'T KNOW/REMEMBER<br>لا أعرف / تذكر...8<br>DOES NOT WISH TO DISCLOSE<br>لا ترغب في الكشف عنها ...9	

<table>
<tr><td>Q22</td><td>During the past 30 days, about how often did you feel worthless?

IF NECESSARY: all, most, some, a little, or none of the time?
في الأيام الـ 30 الأخيرة، كم يوما لم تشعرين بالتقدير؟
(عند الضرورة: طوال الوقت أو في غالبية الوقت أو لبعض الوقت أو لقليل من الوقت أو أبداً؟)</td><td>ALL
طوال الوقت ...1
MOST
في غالبية الوقت ...2
SOME
لبعض الوقت ...3
A LITTLE
لقليل من الوقت ...4
NONE
أبداً ...5

DOESN'T KNOW/REMEMBER
لا أعرف / تذكر...8
DOES NOT WISH TO DISCLOSE
لا ترغب في الكشف عنها ...9</td><td></td></tr>
<tr><td>Q23</td><td>The last set of questions asked about feelings that may have occurred during the past 30 days.

Taking them altogether, did these feelings occur more often in the past 30 days than is usual for you, about the same as usual, or less often than usual?

تعلقت الأسئلة في المجموعة الأخيرة بالمشاعر التي قد تكونين شعرت بها في الـ30 يوماً الأخيرة. هل خالجتك هذه المشاعر مجتمعة أكثر من المعتاد بالنسبة لك في الـ 30 يوماً الأخيرة، أو كالمعتاد أو أقل من المعتاد؟"</td><td>ALL
طوال الوقت ...1
MOST
في غالبية الوقت ...2
SOME
لبعض الوقت ...3
A LITTLE
لقليل من الوقت ...4
NONE
أبداً ...5

DOESN'T KNOW/REMEMBER
لا أعرف / تذكر...8
DOES NOT WISH TO DISCLOSE
لا ترغب في الكشف عنها ...9</td><td></td></tr>
<tr><td>Q24</td><td>The next questions are about how these feelings may have affected you in the past 30 days.

How many days out of the past 30 were you totally unable to work or carry out your normal activities because of these feelings? (if don't know, put 98, if refused put 99)

"تتعلق الأسئلة التالية بالطريقة التي أثرت بها هذه المشاعر عليك في الـ30 يوماً الاخيرة.
خلال الـ 30 يوماً الأخيرة، كم يوماً شعرت بأنك غير قادرة على العمل أو إنجاز مهامك العادية بسبب هذه المشاعر؟
إذا لم تكن تعرف ، وضعت 98 ، إذا رفضت " (وضع 99)</td><td>NUMBER [ ]
رقم

DOESN'T KNOW/REMEMBER
لا أعرف / تذكر...98
DOES NOT WISH TO DISCLOSE
لا ترغب في الكشف عنها ...99</td><td></td></tr>
</table>

Q25	How many days in the past 30 were you able to do only half or less of what you normally have been able to do because of these feelings? (if don't know, put 98, if refused put 99)<br>كم يوماً طوال الـ 30 يوماً الأخيرة تمكنت من " القيام بنصف ما تقومين به عادةً أو بأقل مما تقومين به عادةً بسبب هذه المشاعر؟	NUMBER [ ]<br>رقم<br><br>DOESN'T KNOW/REMEMBER<br>لا أعرف / تذكر...98<br>DOES NOT WISH TO DISCLOSE<br>لا ترغب في الكشف عنها ...99	
Q26	During the past 30 days, how many times did you see a doctor or other health professional about these feelings? (if don't know, put 98, if refused put 99)<br>إذا كنت لا تعرف ، ضع 98 ، إذا رفضت ، اكتب) 99) في الـ 30 يوماً الأخيرة، كم مرة استشرت طبيباً أو مختصصاً آخر في المجال الصحي بسبب تلك المشاعر؟	NUMBER [ ]<br>رقم<br><br>DOESN'T KNOW/REMEMBER<br>لا أعرف / تذكر...98<br>DOES NOT WISH TO DISCLOSE<br>لا ترغب في الكشف عنها ...99	
Q27	During the past 30 days, how often have physical health problems been the main causes of these feelings - all of the time, most of the time, some of the time, a little of the time, or none of the time?<br>في الأيام الـ 30 الأخيرة، ما كان مدى شعورك بأن مشاكل الصحة الجسدية هي السبب الأساسي لهذه المشاعر؟ - طوال الوقت أو في غالبية الوقت أو لبعض الوقت أو لقليل من الوقت أو أبداً؟	ALL<br>طوال الوقت ...1<br>MOST<br>في غالبية الوقت ...2<br>SOME<br>لبعض الوقت ...3<br>A LITTLE<br>لقليل من الوقت ...4<br>NONE<br>أبداً ...5<br><br>DOESN'T KNOW/REMEMBER<br>لا أعرف / تذكر...8<br>DOES NOT WISH TO DISCLOSE<br>لا ترغب في الكشف عنها ...9	

INTIMATE PARTNER VIOLENCE
العنف من الشريك الحميم

[IF EVER PARTNERED]

[NEVER PARTNERED SKIP TO 67]

I am now going to ask you about some situations that are true for many women. Does your current or most recent husband/partner generally do any of the following?

العنف من الشريك الحميم: سأطرح عليك الآن أسئلة عن بعض الأوضاع التي تعاني منها العديد من النساء. يسأل هذا القسم عن أي تجربة خلال حياتك ، وليس بالضرورة في الوقت الحالي.

أود أن أذكرك بأن أي شيء تقولونه هنا يظل سريًا. لن تتم مشاركة أي من معلوماتك المحددة مع أي شخص. سلامتك وراحتك هي أولويتنا. هل يقوم زوجك/شريكك الحالي بشكل عام بأي مما يلي؟:

ECONOMIC VIOLENCE

[العنف الاقتصادي]

Q28	Prohibits you from getting a job, going to work, earning money or participating in income generating projects?<br>يمنعك من الحصول على وظيفة أو الذهاب إلى العمل أو التجارة أو كسب المال أو المشاركة في مشاريع مدرة للدخل؟	YES<br>نعم...1<br>NO<br>لا...2<br>NO RESPONSE<br>لا أريد الإجابة ...3	
Q29	Takes your money (earnings) from you against your will?<br>يأخذ مالك (المال الذي تكسبينه) منك ضد إرادتك؟	YES<br>نعم...1<br>NO<br>لا...2<br>NO RESPONSE<br>لا أريد الإجابة ...3	
Q30	Refuses to give you money you needed for household expenses even when he has money for other things (such as alcohol and cigarettes)?<br>يرفض إعطاءك المال الذي تحتاجينه لمصاريف المنزل حتى إذا كان لديه المال لأمور أخرى (كالكحول والسجائر))	YES<br>نعم...1<br>NO<br>لا...2<br>NO RESPONSE<br>لا أريد الإجابة ...3	
EMOTIONAL VIOLENCE<br><br>The next questions are about things that happen to many women and that your husband/ partner may have done to you. We are going to be talking about acts done either by your current husband partner OR any previous partner.<br>[العنف العاطفي]<br>تتعلق الأسئلة التالية بأمور تعاني منها العديد من النساء وقد تكونين تعرضت لها من زوجك/شريكك. سوف نتحدث عن الأمور التي قام بها زوجك/شريكك الحالي أو أي شريك سابق. هل قام زوجك/شريكك الحالي أو أي شريك آخر بأي مما يلي...			
Q31	Insulted you or made you feel bad about yourself?<br>إهانتك أو جعلك تشعرين بالسوء؟	YES<br>نعم...1<br>NO<br>لا...2<br>NO RESPONSE<br>لا أريد الإجابة ...3	SKIP TO Q32<br>انتقل إلى السؤال 32
Q31a	In the past 12 months, would you say that this happened once, a few times, or many times?<br>إذا كان الجوب نعم:] في الـ 12 شهراً الماضية،[هل تقولين إن ذلك حدث مرة واحدة أو بضع مرات أو عدة مرات؟	ONCE<br>مرة واحدة ...1<br>FEW TIMES<br>بضع مرات ...2<br>MANY TIMES<br>عدة مرات ...3<br>NEVER<br>أبدا ...4<br>NO RESPONSE<br>لا أريد الإجابة ...5	
Q32	Humiliated you in front of other people?<br>إهانتك أمام أشخاص آخرين؟	YES<br>نعم...1	

		NO<br>لا...2<br>NO RESPONSE<br>لا أريد الإجابة ...3	SKIP TO Q33<br>انتقل إلى السؤال 33
Q32a	In the past 12 months, would you say that this happened once, a few times, or many times?<br>إذا كان الجوب نعم:] في الـ 12 شهراً الماضية،[هل تقولين إن ذلك حدث مرة واحدة أو بضع مرات أو عدة مرات؟	ONCE<br>مرة واحدة ...1<br>FEW TIMES<br>بضع مرات ...2<br>MANY TIMES<br>عدة مرات ...3<br>NEVER<br>أبدا ...4<br>NO RESPONSE<br>لا أريد الإجابة ...5	
Q33	Done things to scare or intimidate you on purpose?<br>إخافتك أو ترهيبك عن قصد؟	YES<br>نعم...1<br>NO<br>لا...2<br>NO RESPONSE<br>لا أريد الإجابة ...3	SKIP TO Q34<br>انتقل إلى السؤال 34
Q33a	In the past 12 months, would you say that this happened once, a few times, or many times?<br>إذا كان الجوب نعم:] في الـ 12 شهراً الماضية،[هل تقولين إن ذلك حدث مرة واحدة أو بضع مرات أو عدة مرات؟	ONCE<br>مرة واحدة ...1<br>FEW TIMES<br>بضع مرات ...2<br>MANY TIMES<br>عدة مرات ...3<br>NEVER<br>أبدا ...4<br>NO RESPONSE<br>لا أريد الإجابة ...5	
Q34	Verbally threatened to hurt you or someone you care about?<br>د) تهديدك شفهياً بأنه سيؤذيك أو يؤذي شخصاً يهمك أمره؟	YES<br>نعم...1<br>NO<br>لا...2<br>NO RESPONSE<br>لا أريد الإجابة ...3	SKIP TO Q35<br>انتقل إلى السؤال 35
Q34a	In the past 12 months, would you say that this happened once, a few times, or many times?<br>إذا كان الجوب نعم:] في الـ 12 شهراً الماضية،[هل تقولين إن ذلك حدث مرة واحدة أو بضع مرات أو عدة مرات؟	ONCE<br>مرة واحدة ...1<br>FEW TIMES<br>بضع مرات ...2<br>MANY TIMES<br>عدة مرات ...3<br>NEVER<br>أبدا ...4<br>NO RESPONSE<br>لا أريد الإجابة ...5	

PHYSICAL VIOLENCE

العنف الجسدي] هل قام زوجك/شريكك أو أي شريك آخر بأي شيء مما يلي] :

Q35	Slapped or thrown something at your that could hurt you?<br>صفعك أو رمي شيئاً قد يؤذيك عليك؟	YES<br>نعم...1<br>NO<br>لا...2<br>NO RESPONSE<br>لا أريد الإجابة ...3	□SKIP TO Q36<br>انتقل إلى السؤال 36
Q35a	In the past 12 months, would you say that this happened once, a few times, or many times?<br>إذا كان الجوب نعم:] في الـ 12 شهراً الماضية،[<br>هل تقولين إن ذلك حدث مرة واحدة أو بضع مرات أو عدة مرات؟	ONCE<br>مرة واحدة ...1<br>FEW TIMES<br>بضع مرات ...2<br>MANY TIMES<br>عدة مرات ...3<br>NEVER<br>أبدا ...4<br>NO RESPONSE<br>لا أريد الإجابة ...5	
Q36	Pushed you or shoved you or pulled your hair?<br>دفعك أو شد شعرك؟	YES<br>نعم...1<br>NO<br>لا...2<br>NO RESPONSE<br>لا أريد الإجابة ...3	□SKIP TO Q37<br>انتقل إلى السؤال 37
Q36a	In the past 12 months, would you say that this happened once, a few times, or many times?<br>إذا كان الجوب نعم:] في الـ 12 شهراً الماضية،[<br>هل تقولين إن ذلك حدث مرة واحدة أو بضع مرات أو عدة مرات؟	ONCE<br>مرة واحدة ...1<br>FEW TIMES<br>بضع مرات ...2<br>MANY TIMES<br>عدة مرات ...3<br>NEVER<br>أبدا ...4<br>NO RESPONSE<br>لا أريد الإجابة ...5	
Q37	Hit you?<br>ضربك؟	YES<br>نعم...1<br>NO<br>لا...2<br>NO RESPONSE<br>لا أريد الإجابة ...3	□SKIP TO Q38<br>انتقل إلى السؤال 38
Q37a	In the past 12 months, would you say that this happened once, a few times, or many times?<br>إذا كان الجوب نعم:] في الـ 12 شهراً الماضية،[<br>هل تقولين إن ذلك حدث مرة واحدة أو بضع مرات أو عدة مرات؟	ONCE<br>مرة واحدة ...1<br>FEW TIMES<br>بضع مرات ...2<br>MANY TIMES	

		عدة مرات ...3<br>NEVER<br>أبدا ...4<br>NO RESPONSE<br>لا أريد الإجابة ...5	
Q38	Kicked you or dragged you?<br>ركلك أو جرّك؟	YES<br>نعم...1<br>NO<br>لا...2<br>NO RESPONSE<br>لا أريد الإجابة ...3	SKIP TO Q39<br>انتقل إلى السؤال 39
Q38a	In the past 12 months, would you say that this happened once, a few times, or many times?<br>إذا كان الجوب نعم:] في الـ 12 شهراً الماضية،[هل تقولين إن ذلك حدث مرة واحدة أو بضع مرات أو عدة مرات؟	ONCE<br>مرة واحدة ...1<br>FEW TIMES<br>بضع مرات ...2<br>MANY TIMES<br>عدة مرات ...3<br>NEVER<br>أبدا ...4<br>NO RESPONSE<br>لا أريد الإجابة ...5	
Q39	Choked or burnt you on purpose?<br>محاولة خنقك أو إحراقك عن قصد؟	YES<br>نعم...1<br>NO<br>لا...2<br>NO RESPONSE<br>لا أريد الإجابة ...3	SKIP TO Q40<br>انتقل إلى السؤال 40
Q39a	In the past 12 months, would you say that this happened once, a few times, or many times?<br>إذا كان الجوب نعم:] في الـ 12 شهراً الماضية،[هل تقولين إن ذلك حدث مرة واحدة أو بضع مرات أو عدة مرات؟	ONCE<br>مرة واحدة ...1<br>FEW TIMES<br>بضع مرات ...2<br>MANY TIMES<br>عدة مرات ...3<br>NEVER<br>أبدا ...4<br>NO RESPONSE<br>لا أريد الإجابة ...5	
Q40	Threatened or used a gun, knife or other weapon against you?<br>تهديدك أو استخدام مسدس أو سكين أو سلاح آخر ضدك؟	YES<br>نعم...1<br>NO<br>لا...2<br>NO RESPONSE<br>لا أريد الإجابة ...3	SKIP TO Q41<br>انتقل إلى السؤال 41

Q40a	In the past 12 months, would you say that this happened once, a few times, or many times?<br>إذا كان الجوب نعم:] في الـ 12 شهراً الماضية،[هل تقولين إن ذلك حدث مرة واحدة أو بضع مرات أو عدة مرات؟	ONCE<br>مرة واحدة ...1<br>FEW TIMES<br>بضع مرات ...2<br>MANY TIMES<br>عدة مرات ...3<br>NEVER<br>أبدا ...4<br>NO RESPONSE<br>لا أريد الإجابة ...5	
SEXUAL VIOLENCE<br>العنف الجنسي] "هل قام زوجك/شريكك أو أي شريك آخر بأي شيء مما يلي] : ...			
Q41	Did your current husband/partner or any other husband/partner ever force you to have sex when you did not want to?<br>هل أجبرك زوجك / شريكك الحالي أو الأخير على ممارسة الجنس ضد إرادتك؟	YES<br>نعم...1<br>NO<br>لا...2<br>NO RESPONSE<br>لا أريد الإجابة ...3	SKIP TO Q42<br>انتقل إلى السؤال 42
Q41a	In the past 12 months, would you say that this happened once, a few times, or many times?<br>إذا كان الجوب نعم:] في الـ 12 شهراً الماضية،[هل تقولين إن ذلك حدث مرة واحدة أو بضع مرات أو عدة مرات؟	ONCE<br>مرة واحدة ...1<br>FEW TIMES<br>بضع مرات ...2<br>MANY TIMES<br>عدة مرات ...3<br>NEVER<br>أبدا ...4<br>NO RESPONSE<br>لا أريد الإجابة ...5	
Q42	Did you ever have sex when you didn't want to because you were afraid of what your husband/partner might do if you refused?<br>هل مارست الجنس وأنت لا تريدين ذلك لأنك شعرت بالخوف مما قد يفعله زوجك/شريكك إن رفضت؟	YES<br>نعم...1<br>NO<br>لا...2<br>NO RESPONSE<br>لا أريد الإجابة ...3	SKIP TO Q43<br>انتقل إلى السؤال 43
Q42a	In the past 12 months, would you say that this happened once, a few times, or many times?<br>إذا كان الجوب نعم:] في الـ 12 شهراً الماضية،[هل تقولين إن ذلك حدث مرة واحدة أو بضع مرات أو عدة مرات؟	ONCE<br>مرة واحدة ...1<br>FEW TIMES<br>بضع مرات ...2<br>MANY TIMES<br>عدة مرات ...3<br>NEVER<br>أبدا ...4<br>NO RESPONSE<br>لا أريد الإجابة ...5	
Q43	Did your husband/partner or any other husband or partner ever force you to do anything else sexual that you did not want or that you found degrading or humiliating?	YES<br>نعم...1<br>NO	SKIP TO 44

	ج) هل أجبرك زوجك / شريكك الحالي أو الأخيرعلى القيام بأمور جنسية أخرى لم ترغبي في القيام بها أو اعتبرتها مهينة؟	لا...2<br>NO RESPONSE<br>لا أريد الإجابة...3	انتقل إلى السؤال 44
Q43a	In the past 12 months, would you say that this happened once, a few times, or many times?<br>إذا كان الجوب نعم:] في الـ 12 شهراً الماضية،[هل تقولين إن ذلك حدث مرة واحدة أو بضع مرات أو عدة مرات؟	ONCE<br>مرة واحدة... 1<br>FEW TIMES<br>بضع مرات ...2<br>MANY TIMES<br>عدة مرات ...3<br>NEVER<br>أبدا ...4<br>NO RESPONSE<br>لا أريد الإجابة... 5	
**NON-PARTNER VIOLENCE**			
[ALL WOMEN]<br>In your lifetime has anyone (except any husband/male partner) ever done any of the following things to you? This could be by anybody, for example someone you have known such as relatives, neighbours, but also strangers, military and so on.<br>العنف الجنسي الممارَس من غير الشريك<br>طوال حياتك هل تعرضت لأي من الأمور التالية من شخص ما(باستثناء أي زوج/شريك ذكر)؟ قد يكون هذا الشخص أياً كان، كشخص تعرفينه من أقاربك أو جيرانك أو شخص غريب أو جندي إلخ... هل قام شخص ما بأي مما يلي...			
Q44	Beaten you, kicked you, or hurt you with a stick or other object?<br>ضربك أو ركلك أو إيذائك بعصا أو أداة أخرى؟	YES<br>نعم...1<br>NO<br>لا...2<br>NO RESPONSE<br>لا أريد الإجابة... 3	□SKIP TO Q45<br>انتقل إلى السؤال 45
Q44a	In the past 12 months, would you say that this happened once, a few times, or many times?<br>إذا كان الجوب نعم:] في الـ 12 شهراً الماضية،[هل تقولين إن ذلك حدث مرة واحدة أو بضع مرات أو عدة مرات؟	ONCE<br>مرة واحدة... 1<br>FEW TIMES<br>بضع مرات ...2<br>MANY TIMES<br>عدة مرات ...3<br>NEVER<br>أبدا ...4<br>NO RESPONSE<br>لا أريد الإجابة... 5	
Q44b	Who was, or who were the persons that did this to you? Please just tell us in general who did this to you (e.g. family member, teacher, member of the military) and not specific names. Remember, to include people known to you as well as strangers.<br>*(Select all that apply)*	FATHER/STEPFATHER(MOTHER'S HUSBAND<br>الأب/زوج الأم ...A<br>MOTHER/STEPMOTHER(FATHER'S WIFE)<br>الأم/زوجة الأب ...B<br>FATHER-IN-LAW<br>والد الزوج/الزوجة ...C<br>MOTHER-IN-LAW<br>والدة الزوج/الزوجة ...D	

	إذا كان الجواب نعم، من كان الشخص أو الأشخاص الذين فعلوا ذلك بك؟ أخبرينا رجاءً بشكل عام من فعل ذلك بك (مثلاً أحد أفراد العائلة، مدرّس، جندي) من دون أن تكشفي عن اسمه. تذكري أن تشملي أيضاً الأشخاص الذين هم غرباء بالنسبة لك (أجيبي باستخدام الرموز في العمود التالي). في الجهاز اللوحي، يتعين إتاحة خيارات عديدة (وإذا اختارت المشاركات ""غير ذلك"" يمكن إضافة خيار للتحديد).	OTHER MALE FAMILY MEMBER<br>(فرد آخر من العائلة (ذكر …E<br>OTHER FEMALE FAMILY MEMBER<br>(فرد آخر من العائلة (أنثى …F<br>MALE FRIEND/NEIGHBOR/OTHER KNOWN INDIVIDUAL<br>(صديق/جار/زميل في الصف/فرد آخر معروف (ذكر …G<br>FEMALE FRIEND/NEIGHBOR/OTHER KNOWN INDIVIDUAL<br>(صديقة/جارة/زميلة في الصف/فرد آخر معروف (ذكر …H<br>MALE POLICE<br>شرطيّ …I<br>FEMALE POLICE<br>شرطية …J<br>MALE ARMED ACTOR<br>(شخص مسلح (ذكر …K<br>FEMALE ARMED ACTOR<br>(شخص مسلح (أنثى …L<br>MALE OTHER COMMUNITY/ NATIONALITY/ETHNIC GROUP<br>فرد من مجتمع آخر/جنسية/مجموعة إثنية (أخرى (ذكر …M<br>FEMALE OTHER COMMUNITY/ NATIONALITY/ETHNIC GROUP<br>فرد من مجتمع آخر/جنسية/مجموعة إثنية (أخرى (أنثى …N<br>TEACHER (MALE)<br>مدرّس …O<br>TEACHER (FEMALE)<br>مدرّسة …P<br>MALE HUMANITARIAN WORKER<br>عامل إغاثة إنسانية …Q<br>FEMALE HUMANITARIAN WORKER<br>عاملة إغاثة إنسانية …R<br>MALE STRANGER<br>(شخص غريب (ذكر …S<br>FEMALE STRANGER<br>(شخص غريب (أنثى …T<br>OTHER (Please specify)<br>غير ذلك …X<br><br>DOESN'T KNOW/REMEMBER<br>لا أعرف / تذكر…8<br>DOES NOT WISH TO DISCLOSE<br>لا ترغب في الكشف عنها …9	
Q45	Threatened you or actually used a gun, knife, machete or other weapon on you?<br>تهديدك أو استعمال مسدس أو سكين أو منجل أو سلاح آخر ضدك؟	YES<br>نعم…1<br>NO<br>لا…2	 <br><br>**SKIP TO 46**

		NO RESPONSE<br>لا أريد الإجابة ...3	46<br>انتقل إلى السؤال
Q45a	In the past 12 months, would you say that this happened once, a few times, or many times?<br>إذا كان الجوب نعم:] في الـ 12 شهراً الماضية،[هل تقولين إن ذلك حدث مرة واحدة أو بضع مرات أو عدة مرات؟	ONCE<br>مرة واحدة ...1<br>FEW TIMES<br>بضع مرات ...2<br>MANY TIMES<br>عدة مرات ...3<br>NEVER<br>أبدا ...4<br>NO RESPONSE<br>لا أريد الإجابة ...5	
Q45b	Who was, or who were the persons that did this to you? Please just tell us in general who did this to you (e.g. family member, teacher, member of the military) and not specific names. Remember, to include people known to you as well as strangers.<br><br>*(Select all that apply)*<br><br>إذا كان الجواب نعم، من كان الشخص أو الأشخاص الذين فعلوا ذلك بك؟ أخبرينا رجاءً بشكل عام من فعل ذلك بك (مثلاً أحد أفراد العائلة، مدرّس، جندي) من دون أن تكشفي عن اسمه. تذكري أن تشملي أيضاً الأشخاص الذين هم غرباء بالنسبة لك (أجيبي باستخدام الرموز في العمود التالي). في الجهاز اللوحي، يتعين إتاحة خيارات عديدة (وإذا اختارت المشاركات ""غير ذلك"" يمكن إضافة خيار للتحديد).	FATHER/STEPFATHER(MOTHER'S HUSBAND<br>الأب/زوج الأم ...A<br>MOTHER/STEPMOTHER(FATHER'S WIFE)<br>الأم/زوجة الأب ...B<br>FATHER-IN-LAW<br>والد الزوج/الزوجة ...C<br>MOTHER-IN-LAW<br>والدة الزوج/الزوجة ...D<br>OTHER MALE FAMILY MEMBER<br>(فرد آخر من العائلة (ذكر ...E<br>OTHER FEMALE FAMILY MEMBER<br>(فرد آخر من العائلة (أنثى ...F<br>MALE FRIEND/NEIGHBOR/OTHER KNOWN INDIVIDUAL<br>(صديق/جار/زميل في الصف/فرد آخر معروف (ذكر ...G<br>FEMALE FRIEND/NEIGHBOR/OTHER KNOWN INDIVIDUAL<br>(صديقة/جارة/زميلة في الصف/فرد آخر معروف (ذكر ...H<br>MALE POLICE<br>شرطيّ ...I<br>FEMALE POLICE<br>شرطية ...J<br>MALE ARMED ACTOR<br>(شخص مسلح (ذكر ...K<br>FEMALE ARMED ACTOR<br>(شخص مسلح (أنثى ...L<br>MALE OTHER COMMUNITY/ NATIONALITY/ETHNIC GROUP<br>فرد من مجتمع آخر/جنسية/مجموعة إثنية أخرى (ذكر ...M<br>FEMALE OTHER COMMUNITY/ NATIONALITY/ETHNIC GROUP<br>فرد من مجتمع آخر/جنسية/مجموعة إثنية أخرى (أنثى ...N<br>TEACHER (MALE)	

		O… مدرّس<br>TEACHER (FEMALE)<br>P… مدرّسة<br>MALE HUMANITARIAN WORKER<br>Q… عامل إغاثة إنسانية<br>FEMALE HUMANITARIAN WORKER<br>R… عاملة إغاثة إنسانية<br>MALE STRANGER<br>S… (شخص غريب (ذكر<br>FEMALE STRANGER<br>T… (شخص غريب (أنثى<br>OTHER (Please specify)<br>X… غير ذلك<br><br>DOESN'T KNOW/REMEMBER<br>لا أعرف / تذكر…8<br>DOES NOT WISH TO DISCLOSE<br>لا ترغب في الكشف عنها …9	
Q46	Forced you to undress or stripped off your clothing?<br>إجبارك على خلع ملابسك أو خلع ملابسك بنفسه؟	YES<br>نعم…1<br>NO<br>لا…2<br>NO RESPONSE<br>لا أريد الإجابة …3	SKIP TO Q47<br>انتقل إلى السؤال 47
Q46a	In the past 12 months, would you say that this happened once, a few times, or many times?<br>إذا كان الجوب نعم: [في الـ 12 شهراً الماضية،] هل تقولين إن ذلك حدث مرة واحدة أو بضع مرات أو عدة مرات؟	ONCE<br>مرة واحدة …1<br>FEW TIMES<br>بضع مرات …2<br>MANY TIMES<br>عدة مرات …3<br>NEVER<br>أبدا …4<br>NO RESPONSE<br>لا أريد الإجابة …5	
Q46b	Who was, or who were the persons that did this to you? Please just tell us in general who did this to you (e.g. family member, teacher, member of the military) and not specific names. Remember, to include people known to you as well as strangers.<br><br>*(Select all that apply)*<br><br>إذا كان الجواب نعم، من كان الشخص أو الأشخاص الذين فعلوا ذلك بك؟ أخبرينا رجاءً بشكل عام من فعل ذلك بك (مثلاً أحد أفراد العائلة، مدرّس، جندي) من دون أن تكشفي عن اسمه. تذكري أن تشملي أيضاً الأشخاص الذين هم غرباء بالنسبة لك (أجيبي باستخدام الرموز في العمود	FATHER/STEPFATHER(MOTHER'S HUSBAND<br>A… الأب/زوج الأم<br>MOTHER/STEPMOTHER(FATHER'S WIFE)<br>B… الأم/زوجة الأب<br>FATHER-IN-LAW<br>C… والد الزوج/الزوجة<br>MOTHER-IN-LAW<br>D… والدة الزوج/الزوجة<br>OTHER MALE FAMILY MEMBER<br>E… (فرد آخر من العائلة (ذكر<br>OTHER FEMALE FAMILY MEMBER<br>F… (فرد آخر من العائلة (أنثى<br>MALE FRIEND/NEIGHBOR/OTHER KNOWN INDIVIDUAL	

	التالي). في الجهاز اللوحي، يتعين إتاحة خيارات عديدة (وإذا اختارت المشاركات ""غير ذلك"" يمكن إضافة خيار للتحديد).	صديق/جار/زميل في الصف/فرد آخر معروف (ذكر) …G<br>FEMALE FRIEND/NEIGHBOR/OTHER KNOWN INDIVIDUAL<br>(صديقة/جارة/زميلة في الصف/فرد آخر معروف (ذكر …H<br>MALE POLICE<br>شرطيّ …I<br>FEMALE POLICE<br>شرطية …J<br>MALE ARMED ACTOR<br>(شخص مسلح (ذكر …K<br>FEMALE ARMED ACTOR<br>(شخص مسلح (أنثى …L<br>MALE OTHER COMMUNITY/ NATIONALITY/ETHNIC GROUP<br>فرد من مجتمع آخر/جنسية/مجموعة إثنية أخرى (ذكر) …M<br>FEMALE OTHER COMMUNITY/ NATIONALITY/ETHNIC GROUP<br>فرد من مجتمع آخر/جنسية/مجموعة إثنية أخرى (أنثى) …N<br>TEACHER (MALE)<br>مدرّس …O<br>TEACHER (FEMALE)<br>مدرّسة …P<br>MALE HUMANITARIAN WORKER<br>عامل إغاثة إنسانية …Q<br>FEMALE HUMANITARIAN WORKER<br>عاملة إغاثة إنسانية …R<br>MALE STRANGER<br>(شخص غريب (ذكر …S<br>FEMALE STRANGER<br>(شخص غريب (أنثى …T<br>OTHER (Please specify)<br>غير ذلك …X<br><br>DOESN'T KNOW/REMEMBER<br>لا أعرف / تذكر…8<br>DOES NOT WISH TO DISCLOSE<br>لا ترغب في الكشف عنها …9	
Q47	Forced you into having sex when you did not want it, for example by threatening you, holding you down, or putting you in a situation where you could not say no?<br>إجبارك على ممارسة الجنس ضد إرادتك مثلاً بواسطة التهديد أو الضغط عليك أو وضعك في موقف لا يمكنك فيه الرفض؟	YES<br>نعم…1<br>NO<br>لا…2<br>NO RESPONSE<br>لا أريد الإجابة …3	**SKIP TO Q47**<br>**انتقل إلى السؤال 47**

Q47a	In the past 12 months, would you say that this happened once, a few times, or many times?<br>إذا كان الجوب نعم: *[* في الـ *12* شهراً الماضية، *[*<br>هل تقولين إن ذلك حدث مرة واحدة أو بضع مرات أو عدة مرات؟	ONCE<br>مرة واحدة ...1<br>FEW TIMES<br>بضع مرات ...2<br>MANY TIMES<br>عدة مرات ...3<br>NEVER<br>أبدا ...4<br>NO RESPONSE<br>لا أريد الإجابة ...5	
Q47b	Who was, or who were the persons that did this to you? Please just tell us in general who did this to you (e.g. family member, teacher, member of the military) and not specific names. Remember, to include people known to you as well as strangers.<br><br>*(Select all that apply)*<br><br>إذا كان الجواب نعم، من كان الشخص أو الأشخاص الذين فعلوا ذلك بك؟ أخبرينا رجاءً بشكل عام من فعل ذلك بك (مثلاً أحد أفراد العائلة، مدرّس، جندي) من دون أن تكشفي عن اسمه. تذكري أن تشملي أيضاً الأشخاص الذين هم غرباء بالنسبة لك (أجيبي باستخدام الرموز في العمود التالي). في الجهاز اللوحي، يتعين إتاحة خيارات عديدة (وإذا اختارت المشاركات ""غير ذلك"" يمكن إضافة خيار للتحديد).	FATHER/STEPFATHER(MOTHER'S HUSBAND<br>الأب/زوج الأم ...A<br>MOTHER/STEPMOTHER(FATHER'S WIFE)<br>الأم/زوجة الأب ...B<br>FATHER-IN-LAW<br>والد الزوج/الزوجة ...C<br>MOTHER-IN-LAW<br>والدة الزوج/الزوجة ...D<br>OTHER MALE FAMILY MEMBER<br>(فرد آخر من العائلة (ذكر ...E<br>OTHER FEMALE FAMILY MEMBER<br>(فرد آخر من العائلة (أنثى ...F<br>MALE FRIEND/NEIGHBOR/OTHER KNOWN INDIVIDUAL<br>(صديق/جار/زميل في الصف/فرد آخر معروف (ذكر ...G<br>FEMALE FRIEND/NEIGHBOR/OTHER KNOWN INDIVIDUAL<br>(صديقة/جارة/زميلة في الصف/فرد آخر معروف (ذكر ...H<br>MALE POLICE<br>شرطيّ ...I<br>FEMALE POLICE<br>شرطية ...J<br>MALE ARMED ACTOR<br>(شخص مسلح (ذكر ...K<br>FEMALE ARMED ACTOR<br>(شخص مسلح (أنثى ...L<br>MALE OTHER COMMUNITY/ NATIONALITY/ETHNIC GROUP<br>فرد من مجتمع آخر/جنسية/مجموعة إثنية أخرى (ذكر ...M<br>FEMALE OTHER COMMUNITY/ NATIONALITY/ETHNIC GROUP<br>فرد من مجتمع آخر/جنسية/مجموعة إثنية أخرى (أنثى ...N<br>TEACHER (MALE)<br>مدرّس ...O<br>TEACHER (FEMALE)<br>مدرّسة ...P	

		MALE<br>HUMANITARIAN WORKER<br>Q… عامل إغاثة إنسانية<br>FEMALE HUMANITARIAN WORKER<br>R… عاملة إغاثة إنسانية<br>MALE STRANGER<br>S… (شخص غريب (ذكر<br>FEMALE STRANGER<br>T… (شخص غريب (أنثى<br>OTHER (Please specify)<br>X… غير ذلك<br><br>DOESN'T KNOW/REMEMBER<br>لا أعرف / تذكر…8<br>DOES NOT WISH TO DISCLOSE<br>لا ترغب في الكشف عنها …9	
Q48	Attempted to force you into sex (which did not take place)?<br>محاولة إجبارك على ممارسة الجنس (ولم تمارسا الجنس)	YES<br>نعم…1<br>NO<br>لا…2<br>NO RESPONSE<br>لا أريد الإجابة …3	SKIP TO Q49<br>انتقل إلى السؤال 49
Q48a	In the past 12 months, would you say that this happened once, a few times, or many times?<br>إذا كان الجوب نعم:] في الـ 12 شهراً الماضية،[هل تقولين إن ذلك حدث مرة واحدة أو بضع مرات أو عدة مرات؟	ONCE<br>مرة واحدة …1<br>FEW TIMES<br>بضع مرات …2<br>MANY TIMES<br>عدة مرات …3<br>NEVER<br>أبدا …4<br>NO RESPONSE<br>لا أريد الإجابة …5	
Q48b	Who was, or who were the persons that did this to you? Please just tell us in general who did this to you (e.g. family member, teacher, member of the military) and not specific names. Remember, to include people known to you as well as strangers.<br><br>*(Select all that apply)*<br><br>إذا كان الجواب نعم، من كان الشخص أو الأشخاص الذين فعلوا ذلك بك؟ أخبرينا رجاءً بشكل عام من فعل ذلك بك (مثلاً أحد أفراد العائلة، مدرّس، جندي) من دون أن تكشفي عن اسمه. تذكري أن تشملي أيضاً الأشخاص الذين هم غرباء بالنسبة لك (أجيبي باستخدام الرموز في العمود التالي). في الجهاز اللوحي، يتعين إتاحة خيارات	FATHER/STEPFATHER(MOTHER'S HUSBAND<br>A… الأب/زوج الأم<br>MOTHER/STEPMOTHER(FATHER'S WIFE)<br>B… الأم/زوجة الأب<br>FATHER-IN-LAW<br>C… والد الزوج/الزوجة<br>MOTHER-IN-LAW<br>D… والدة الزوج/الزوجة<br>OTHER MALE FAMILY MEMBER<br>E… (فرد آخر من العائلة (ذكر<br>OTHER FEMALE FAMILY MEMBER<br>F… (فرد آخر من العائلة (أنثى<br>MALE FRIEND/NEIGHBOR/OTHER KNOWN INDIVIDUAL<br>(صديق/جار/زميل في الصف/فرد آخر معروف (ذكر<br>…G<br>FEMALE FRIEND/NEIGHBOR/OTHER	

	عديدة (وإذا اختارت المشاركات ""غير ذلك"" يمكن إضافة خيار للتحديد).	KNOWN INDIVIDUAL (صديقة/جارة/زميلة في الصف/فرد آخر معروف (ذكر …H<br>MALE POLICE<br>شرطيّ …I<br>FEMALE POLICE<br>شرطية …J<br>MALE ARMED ACTOR<br>(شخص مسلح (ذكر …K<br>FEMALE ARMED ACTOR<br>(شخص مسلح (أنثى …L<br>MALE OTHER COMMUNITY/ NATIONALITY/ETHNIC GROUP<br>فرد من مجتمع آخر/جنسية/مجموعة إثنية أخرى (ذكر …M<br>FEMALE OTHER COMMUNITY/ NATIONALITY/ETHNIC GROUP<br>فرد من مجتمع آخر/جنسية/مجموعة إثنية أخرى (أنثى …N<br>TEACHER (MALE)<br>مدرّس …O<br>TEACHER (FEMALE)<br>مدرّسة …P<br>MALE HUMANITARIAN WORKER<br>عامل إغاثة إنسانية …Q<br>FEMALE HUMANITARIAN WORKER<br>عاملة إغاثة إنسانية …R<br>MALE STRANGER<br>(شخص غريب (ذكر …S<br>FEMALE STRANGER<br>(شخص غريب (أنثى …T<br>OTHER (Please specify)<br>غير ذلك …X<br><br>DOESN'T KNOW/REMEMBER<br>لا أعرف / تذكر…8<br>DOES NOT WISH TO DISCLOSE<br>لا ترغب في الكشف عنها …9	
Q49	Touched you sexually or did anything else sexually that you did not want to do?<br>التحرش بك جنسياً أو القيام بحركات جنسية أخرى لم ترغبي بها؟	YES<br>نعم…1<br>NO<br>لا…2<br>NO RESPONSE<br>لا أريد الإجابة …3	SKIP TO Q50<br>انتقل إلى السؤال 50
Q49a	In the past 12 months, would you say that this happened once, a few times, or many times?<br>إذا كان الجوب نعم:] في الـ 12 شهراً الماضية،[هل تقولين إن ذلك حدث مرة واحدة أو بضع مرات أو عدة مرات؟	ONCE<br>مرة واحدة …1<br>FEW TIMES<br>بضع مرات …2<br>MANY TIMES<br>عدة مرات …3<br>NEVER	

		أبدا ...4<br>NO RESPONSE<br>لا أريد الإجابة ...5	
Q49b	Who was, or who were the persons that did this to you? Please just tell us in general who did this to you (e.g. family member, teacher, member of the military) and not specific names. Remember, to include people known to you as well as strangers.<br><br>*(Select all that apply)*<br><br>إذا كان الجواب نعم، من كان الشخص أو الأشخاص الذين فعلوا ذلك بك؟ أخبرينا رجاءً بشكل عام من فعل ذلك بك (مثلاً أحد أفراد العائلة، مدرّس، جندي) من دون أن تكشفي عن اسمه. تذكري أن تشملي أيضاً الأشخاص الذين هم غرباء بالنسبة لك (أجيبي باستخدام الرموز في العمود التالي). في الجهاز اللوحي، يتعين إتاحة خيارات عديدة (وإذا اختارت المشاركات ""غير ذلك"" يمكن إضافة خيار للتحديد).	FATHER/STEPFATHER(MOTHER'S HUSBAND<br>الأب/زوج الأم ...A<br>MOTHER/STEPMOTHER(FATHER'S WIFE)<br>الأم/زوجة الأب ...B<br>FATHER-IN-LAW<br>والد الزوج/الزوجة ...C<br>MOTHER-IN-LAW<br>والدة الزوج/الزوجة ...D<br>OTHER MALE FAMILY MEMBER<br>(فرد آخر من العائلة (ذكر ...E<br>OTHER FEMALE FAMILY MEMBER<br>(فرد آخر من العائلة (أنثى ...F<br>MALE FRIEND/NEIGHBOR/OTHER KNOWN INDIVIDUAL<br>(صديق/جار/زميل في الصف/فرد آخر معروف (ذكر<br>...G<br>FEMALE FRIEND/NEIGHBOR/OTHER KNOWN INDIVIDUAL<br>(صديقة/جارة/زميلة في الصف/فرد آخر معروف (ذكر<br>...H<br>MALE POLICE<br>شرطيّ ...I<br>FEMALE POLICE<br>شرطية ...J<br>MALE ARMED ACTOR<br>(شخص مسلح (ذكر ...K<br>FEMALE ARMED ACTOR<br>(شخص مسلح (أنثى ...L<br>MALE OTHER COMMUNITY/ NATIONALITY/ETHNIC GROUP<br>فرد من مجتمع آخر/جنسية/مجموعة إثنية<br>(أخرى (ذكر ...M<br>FEMALE OTHER COMMUNITY/ NATIONALITY/ETHNIC GROUP<br>فرد من مجتمع آخر/جنسية/مجموعة إثنية<br>(أخرى (أنثى ...N<br>TEACHER (MALE)<br>مدرّس ...O<br>TEACHER (FEMALE)<br>مدرّسة ...P<br>MALE HUMANITARIAN WORKER<br>عامل إغاثة إنسانية ...Q<br>FEMALE HUMANITARIAN WORKER<br>عاملة إغاثة إنسانية ...R<br>MALE STRANGER<br>(شخص غريب (ذكر ...S<br>FEMALE STRANGER	

		T… (شخص غريب (أنثى<br>OTHER (Please specify)<br>X… غير ذلك<br><br>DOESN'T KNOW/REMEMBER<br>لا أعرف / تذكر…8<br>DOES NOT WISH TO DISCLOSE<br>لا ترغب في الكشف عنها …9	
Q50	Promised to give you gifts, helped you pay for things or help you in other ways in exchange for sex?<br>وعدك بتقديم الهدايا لك أو مساعدتك على شراء بضعة أمور أو تقديم المساعدة لك بطرق أخرى مقابل الجنس؟	YES<br>نعم…1<br>NO<br>لا…2<br>NO RESPONSE<br>لا أريد الإجابة …3	SKIP TO Q51<br>انتقل إلى السؤال 51
Q50a	In the past 12 months, would you say that this happened once, a few times, or many times?<br>إذا كان الجوب نعم: [في الـ 12 شهراً الماضية، [<br>هل تقولين إن ذلك حدث مرة واحدة أو بضع مرات أو عدة مرات؟	ONCE<br>مرة واحدة …1<br>FEW TIMES<br>بضع مرات …2<br>MANY TIMES<br>عدة مرات …3<br>NEVER<br>أبدا …4<br>NO RESPONSE<br>لا أريد الإجابة …5	
Q50b	Who was, or who were the persons that did this to you? Please just tell us in general who did this to you (e.g. family member, teacher, member of the military) and not specific names. Remember, to include people known to you as well as strangers.<br><br>*(Select all that apply)*<br><br>إذا كان الجواب نعم، من كان الشخص أو الأشخاص الذين فعلوا ذلك بك؟ أخبرينا رجاءً بشكل عام من فعل ذلك بك (مثلاً أحد أفراد العائلة، مدرّس، جندي) من دون أن تكشفي عن اسمه. تذكري أن تشملي أيضاً الأشخاص الذين هم غرباء بالنسبة لك (أجيبي باستخدام الرموز في العمود التالي). في الجهاز اللوحي، يتعين إتاحة خيارات عديدة (وإذا اختارت المشاركات ""غير ذلك"" يمكن إضافة خيار للتحديد).	FATHER/STEPFATHER(MOTHER'S HUSBAND<br>A… الأب/زوج الأم<br>MOTHER/STEPMOTHER(FATHER'S WIFE)<br>B… الأم/زوجة الأب<br>FATHER-IN-LAW<br>C… والد الزوج/الزوجة<br>MOTHER-IN-LAW<br>D… والدة الزوج/الزوجة<br>OTHER MALE FAMILY MEMBER<br>E… (فرد آخر من العائلة (ذكر<br>OTHER FEMALE FAMILY MEMBER<br>F… (فرد آخر من العائلة (أنثى<br>MALE FRIEND/NEIGHBOR/OTHER KNOWN INDIVIDUAL<br>(صديق/جار/زميل في الصف/فرد آخر معروف (ذكر<br>…G<br>FEMALE FRIEND/NEIGHBOR/OTHER KNOWN INDIVIDUAL<br>(صديقة/جارة/زميلة في الصف/فرد آخر معروف (ذكر<br>…H<br>MALE POLICE<br>I… شرطيّ<br>FEMALE POLICE<br>J… شرطية<br>MALE ARMED ACTOR	

		K... (شخص مسلح (ذكر<br>FEMALE ARMED ACTOR<br>L... (شخص مسلح (أنثى<br>MALE OTHER COMMUNITY/ NATIONALITY/ETHNIC GROUP<br>فرد من مجتمع آخر/جنسية/مجموعة إثنية<br>M... (أخرى (ذكر<br>FEMALE OTHER COMMUNITY/ NATIONALITY/ETHNIC GROUP<br>فرد من مجتمع آخر/جنسية/مجموعة إثنية<br>N... (أخرى (أنثى<br>TEACHER (MALE)<br>O... مدرّس<br>TEACHER (FEMALE)<br>P... مدرّسة<br>MALE HUMANITARIAN WORKER<br>Q... عامل إغاثة إنسانية<br>FEMALE HUMANITARIAN WORKER<br>R... عاملة إغاثة إنسانية<br>MALE STRANGER<br>S... (شخص غريب (ذكر<br>FEMALE STRANGER<br>T... (شخص غريب (أنثى<br>OTHER (Please specify)<br>X... غير ذلك<br><br>DOESN'T KNOW/REMEMBER<br>لا أعرف / تذكر...8<br>DOES NOT WISH TO DISCLOSE<br>لا ترغب في الكشف عنها ...9	
**COPING AND HELP SEEKING**<br>**آليات التكيف الإيجابية (جرد COPE)**			
Please read the following out loud: please describe your coping mechanisms for any of the above experiences.<br>.يرجى قراءة ما يلي بصوت عال: يرجى وصف آليات التكيف الإيجابية الخاصة بك لأي من التجارب المذكورة أعلاه			
Q53	I try to get advice from someone about what to do<br>أحاول الحصول على النصح من شخص آخر بشأن ما يجب القيام به.	DISAGREE<br>أعارض ...1<br>NEUTRAL<br>حيادي ...2<br>AGREE<br>أوافق ...3	
Q54	I try to find comfort in my religion<br>أحاول العثور على الراحة في ديني	DISAGREE<br>أعارض ...1<br>NEUTRAL<br>حيادي ...2<br>AGREE<br>أوافق ...3	

Q55	I try to get emotional support from friends or relatives<br>أحاول الحصول على الدعم العاطفي من الأصدقاء أو الأقارب	DISAGREE<br>أعارض ...1<br>NEUTRAL<br>حيادي ...2<br>AGREE<br>أوافق ...3	
Q56	I accept that this has happened and that it can't be changed<br>أتقبل حصول ذلك وأنه لا يمكن تغييره	DISAGREE<br>أعارض ...1<br>NEUTRAL<br>حيادي ...2<br>AGREE<br>أوافق ...3	
HELP SEEKING / ACCESS TO SERVICES<br>الوصول إلى الخدمات			
The following questions are about any services you have accessed in Lebanon:<br>الأسئلة التالية تدور حول أي خدمات قمت بالحصول عليها في لبنان:			
Q57	We have discussed issues about women's health, and about violence.<br><br>Have you ever accessed services to help you address any of these issues?<br>ناقشنا أموراً تتعلق بصحة المرأة وبالعنف. هل سبق أن حصلت على خدمات تساعدك على معالجة أي من هذه المسائل؟	YES<br>نعم...1<br>NO<br>لا...2<br>NO RESPONSE<br>لا أريد الإجابة ...3	**SKIP TO Q60**<br>**انتقل إلى السؤال 60**
Q58	If so, what types of services?<br>ما هم أنواع الخدمات التي حصلت عليها؟	PSYCHOSOCIAL SUPPORT<br>الدعم النفسي الاجتماعي (أنشطة جماعية مرتبطة بالثقة بالنفس والمهارات الحياتية الأساسية) ...1<br>REPRODUCTIVE/MATERNAL HEALTH SERVICES<br>الصحة الإنجابية/صحة الأم ...2<br>MENTAL HEALTH<br>الصحة النفسية ...3<br>OTHER TYPES OF HEALTH SERVICES<br>أنواع أخرى من الخدمات الصحية ...4<br>CASE MANAGEMENT<br>إدارة الحالات ...5<br>EDUCATION<br>التعليم ...6<br>VOCATIONAL TRAINING<br>التدريب المهني ...7<br>LEGAL AID<br>المساعدة القانونية ...8<br>SAFETY/SECURITY<br>السلامة/الأمن ...9<br>CASH<br>النقد ...10	

		OTHER (Please specify)<br>غير ذلك ...11	
Q59	If so, what were the factors that supported you to access services?<br>إذا كان الجواب نعم، ما هي العوامل التي دعمتك في الحصول على الخدمات؟	SERVICES WERE CLOSE TO RESIDENCE<br>كان مكان تقديم الخدمات قريباً من مكان سكني ...1<br>INFORMATION ABOUT SERVICES WAS CLEAR AND ACCESSIBLE<br>كانت المعلومات عن هذه الخدمات واضحة وسهلة ...2<br>SERVICES WERE TARGETED TO PEOPLE OF MY BACKGROUND / STATUS<br>استهدفت هذه الخدمات أشخاصاً من خلفيتي/بوضعي ...3<br>FAMILY MEMBERS/PARTNER WERE SUPPORTIVE<br>دعمني أفراد عائلتي/شريكي في الوصول إلى هذه الخدمات ...4<br>KNEW THESE SERVICES WOULD PROVIDE NEEDED SUPPORT<br>عرفت أن هذه الخدمات ستوفر لي الدعم النفسي والاجتماعي الضروري ...5<br>FELT ACCEPTED & SAFE IN CENTERS & WITH STAFF<br>شعرت بالقبول والأمان في مراكز توفير الخدمات/مع مقدمي الخدمات ...6<br>TRUSTS THE ORGANIZATION OFFERING THE SERVICE(S)<br>أثق بالمنظمة (المنظمات) التي توفر الخدمات 7..	
Q60	If not, what were the reasons that you didn't/could not access services?<br>إذا كان الجواب لا، ما هي الأسباب التي منعتك من الوصول إلى الخدمات؟	DID NOT NED SERVICES<br>لم أكن بحاجة للخدمات ...1<br>DID NOT KNOW ABOUT ANY SERVICES<br>لم أكن أعرف شيئاً عن أي خدمات ...2<br>SERVICES WERE TOO FAR AWAY<br>كانت الخدمات تقدّم في مكان بعيد جداً ...3<br>PEOPLE OF MY BACKGROUND/ STATUS NOT ELIGIBLE<br>الأشخاص من خلفيتي/في وضعي ليسوا مؤهلين ...4<br>FAMILY MEMBERS/PARTNER DID NOT ALLOW<br>أفراد عائلتي/شريكي لم يسمحوا لي بالحصول على الخدمات ...5<br>FEAR OF THREATS/CONSEQUENCES/ MORE VIOLENCE<br>الخوف من التهديد/العواقب/المزيد من العنف...6<br>SITUATION IS NORMAL/NOT SERIOUS<br>الوضع عادي/ليس خطيراً ...7<br>EMBARASSED/ASHAMED/AFRAID	

		SHE WOULD NOT BE BELIEVED OR WOULD BE BLAMED<br>شعرت بالحرج/الخجل/الخوف من أن لا يصدقني أحد أو من أن يلومني الآخرون ...8<br>BELIEVED IT WOULD NOT HELP<br>اعتقدت أن ذلك لن يساعدني...9<br>ORGANIZATIONS NOT HELPFUL IN THE PAST<br>لم تقدم المنظمات أي مساعدة في الماضي ...10	
**PLEASE EXPLAIN:**<br>**يرجى التوضيح (إذا لم يتم تقديم شرح ، اكتب "N / A")**			
Q61	Do you access OTHER services from the UN, humanitarian agencies, NGOs, women's organizations, or community-based organizations?<br>هل حصلت على خدمات أخرى من الأمم المتحدة، أو الوكالات الإنسانية أو المنظمات غير الحكومية أو منظمات المرأة أو المنظمات القائمة على المجتمع؟	YES<br>نعم...1<br>NO<br>لا...2<br>NO RESPONSE<br>لا أريد الإجابة ...3	**SKIP TO Q64**<br>**انتقل إلى السؤال 64**
Q62	If so, what types of services?<br>ما هم أنواع الخدمات التي حصلت عليها؟	PSYCHOSOCIAL SUPPORT<br>الدعم النفسي الاجتماعي (أنشطة جماعية مرتبطة بالثقة بالنفس والمهارات الحياتية الأساسية) ...1<br>REPRODUCTIVE/MATERNAL HEALTH SERVICES<br>الصحة الإنجابية/صحة الأم ...2<br>MENTAL HEALTH<br>الصحة النفسية ...3<br>OTHER TYPES OF HEALTH SERVICES<br>أنواع أخرى من الخدمات الصحية ...4<br>EDUCATION<br>التعليم ...5<br>LEGAL AID<br>المساعدة القانونية ...6<br>SAFETY/SECURITY<br>السلامة/الأمن ...7<br>CASH<br>النقد ...8<br>FOOD<br>الطعام 9 ...<br>BASIC ASSISTANCE/NEEDS<br>المساعدة/الاحتياجات الأساسية ...10<br>SHELTER AND NON-FOOD<br>المأوى والمواد غير الغذائية ...11<br>WATER SANITATION HYGIENE<br>المياه والصحة العامة والنظافة ...12	

Q63	If so, what were the factors that supported you to access services?<br>إذا كان الجواب نعم، ما هي العوامل التي دعمتك في الحصول على الخدمات؟	SERVICES WERE CLOSE TO RESIDENCE<br>كان مكان تقديم الخدمات قريباً من مكان سكني ...1<br>INFORMATION ABOUT SERVICES WAS CLEAR AND ACCESSIBLE<br>كانت المعلومات عن هذه الخدمات واضحة وسهلة ...2<br>SERVICES WERE TARGETED TO PEOPLE OF MY BACKGROUND / STATUS<br>استهدفت هذه الخدمات أشخاصاً من خلفيتي/بوضعي ...3<br>FAMILY MEMBERS/PARTNER WERE SUPPORTIVE<br>دعمني أفراد عائلتي/شريكي في الوصول إلى هذه الخدمات ...4<br>KNEW THESE SERVICES WOULD PROVIDE NEEDED SUPPORT<br>عرفت أن هذه الخدمات ستوفر لي الدعم النفسي والاجتماعي الضروري ...5<br>FELT ACCEPTED & SAFE IN CENTERS & WITH STAFF<br>شعرت بالقبول والأمان في مراكز توفير الخدمات/مع مقدمي الخدمات ...6<br>TRUSTS THE ORGANIZATION OFFERING THE SERVICE(S)<br>أثق بالمنظمة (المنظمات) التي توفر الخدمات 7..	
Q64	If not, what were the reasons that you didn't/could not access services?<br>إذا كان الجواب لا، ما هي الأسباب التي منعتك من الوصول إلى الخدمات؟	DID NOT NED SERVICES<br>لم أكن بحاجة للخدمات ...1<br>DID NOT KNOW ABOUT ANY SERVICES<br>لم أكن أعرف شيئاً عن أي خدمات ...2<br>SERVICES WERE TOO FAR AWAY<br>كانت الخدمات تقدّم في مكان بعيد جداً ...3<br>PEOPLE OF MY BACKGROUND/ STATUS NOT ELIGIBLE<br>الأشخاص من خلفيتي/في وضعي ليسوا مؤهلين ...4<br>FAMILY MEMBERS/PARTNER DID NOT ALLOW<br>أفراد عائلتي/شريكي لم يسمحوا لي بالحصول على الخدمات ...5<br>FEAR OF THREATS/CONSEQUENCES/ MORE VIOLENCE<br>الخوف من التهديد/العواقب/المزيد من العنف...6<br>SITUATION IS NORMAL/NOT SERIOUS<br>الوضع عادي/ليس خطيراً ...7<br>EMBARASSED/ASHAMED/AFRAID SHE WOULD NOT BE BELIEVED OR WOULD BE BLAMED	

		شعرت بالحرج/الخجل/الخوف من أن لا يصدقني أحد أو من أن يلومني الآخرون ...8<br>BELIEVED IT WOULD NOT HELP<br>اعتقدت أن ذلك لن يساعدني...9<br>ORGANIZATIONS NOT HELPFUL IN THE PAST<br>لم تقدم المنظمات أي مساعدة في الماضي ...10	

PLEASE EXPLAIN:

يرجى التوضيح (إذا لم يتم تقديم شرح ، اكتب "N / A")

Did you experience any positive changes as a result of these services?

هل واجهت أي تغييرات إيجابية نتيجة لهذه الخدمات؟

Do you have any comments/questions that you would like to add, or something you thought we did not address in the survey?

هل لديك أي تعليقات / أسئلة ترغب في إضافتها ، أو شيء تعتقد أنه لم يتم تناوله في الاستبيان

I would like to thank you very much for helping us. I appreciate the time that you have taken. I realize that these questions may have been difficult for you to answer, but it is only by hearing from women themselves that we can really understand about their health and experiences related to the war and displacement. From what you have told us, I can tell that you have had some very difficult times in your life. No one has the right to treat someone else in that way. However, from what you have told me I can see also that you are strong, and have survived through some difficult circumstances. If needed, here is a list of organizations that provide support, legal advice and counseling services to women in your community. Please do contact them if you or any of your friends or relatives needs help now or perhaps some time in the future. If you would prefer, I can connect you with people at health and counseling NGOs so that you can talk with them further.Their services are free, and they will keep anything that you say private. [GIVE NAMES OF NGOS FROM YOUR LIST].

" أود أن أشكرك على مساعدتنا وأقدر الوقت الذي خصصته لذلك. أعرف أنه ربما كان من الصعب عليك الإجابة على هذه الأسئلة ولكننا لن نفهم الوضع الصحي للنساء وتجاربهن في ما يتعلق بالحرب والنزوح إلا إذا سمعنا ذلك منهن شخصياً. انطلاقاً مما أخبرتنا به، أستطيع أن أقول لك أنك مررت بأوقات صعبة جداً في حياتك. لا يحق لأحد معاملة شخص آخر بهذه الطريقة. ولكن ما أخبرتني به يظهر أيضاً أنك قوية وأنك نجوت من ظروف صعبة. هذه قائمة بالمنظمات التي توفر الدعم والمشورة القانونية والخدمات الاستشارية للنساء في مجتمعك والتي يمكنك اللجوء إليها عند الحاجة. يرجى منك الاتصال بها إذا كنت أنت أو أي من أصدقائك أو أقاربك بحاجة للمساعدة الآن أو في المستقبل. يمكنني أن أعطيك معلومات للاتصال بأشخاص في منظمات غير حكومية تهتم بالصحة والإرشاد يمكنك التحدث معهم أكثر إن كنت تفضلين ذلك. خدماتهم مجانية وهم سيحافظون على سرية كل ما تقولينه. إذا كنت ترغبين في التحدث إلى أخصائي اجتماعي ، فيرجى الاتصال بالرقم التالي.

+961 1 283 820

81 78 81 78 (safe line)

As we move around your community we are telling people that this is a survey about the health and wellbeing of women and we are not telling anyone who we are interviewing. We know the people in this area will be curious. We suggest that you do not give details about what we discussed, and that you simply say that it is a survey about women. Again thank you for your time.

بينما نتنقل في مجتمعك، نقول للأشخاص إن هذا الاستبيان يتعلق بصحة ورفاه المرأة ولا نخبر أحداً عن هوية الأشخاص الذين نجري معهم المقابلات. نعرف أن الأشخاص في هذه المنطقة فضوليون. ونقترح عليك عدم الكشف عن تفاصيل الأمور التي ناقشناها والقول إن هذا الاستبيان يتعلق بالمرأة.

نشكرك مجدداً على وقتك.
